# Efforts toward PET-Activatable Red-Shifted Silicon Rhodamines and Silicon Pyronine Dyes

**DOI:** 10.3390/ph16030401

**Published:** 2023-03-07

**Authors:** Carsten Sven Kramer, Thines Kanagasundaram, Jessica Matthias, Klaus Kopka

**Affiliations:** 1 Radiopharmaceutical Chemistry, German Cancer Research Center (DKFZ), Im Neuenheimer Feld 223, 69120 Heidelberg, Germany; 2 Institute of Radiopharmaceutical Cancer Research, Helmholtz-Zentrum Dresden-Rossendorf (HZDR) e. V., Bautzner Landstrasse 400, 01328 Dresden, Germany; 3 Institute of Inorganic Chemistry, Heidelberg University, Im Neuenheimer Feld 270, 69120 Heidelberg, Germany; 4 Department of Optical Nanoscopy, Max Planck Institute for Medical Research, 69120 Heidelberg, Germany; 5 Faculty of Chemistry and Food Chemistry, School of Science, Technische Universität Dresden, Mommsenstraße 4, 01069 Dresden, Germany; 6 National Center for Tumor Diseases (NCT) Dresden, University Hospital Carl Gustav Carus, Fetscherstraße 74, 01307 Dresden, Germany; 7 German Cancer Consortium (DKTK), Partner Site Dresden, Fetscherstraße 74, 01307 Dresden, Germany

**Keywords:** tracer, bimodal imaging, PET tracer, fluorine-18, radiofluorination, optical imaging, pyronine, silicon rhodamine, fluorescence dye, near-infrared dye, SiFA-IE

## Abstract

Tracers for bimodal optical imaging and positron emission tomography unite multiple advantages in a single molecule. Their tumor-specific uptake can be visualized after their PET activation by radiofluorination via PET/CT or PET/MRI allowing for staging or therapy planning, while their non-radioactive moiety additionally facilitates the visualization of malignant tissue during intraoperative fluorescence-guided surgery or in histological assessments. The silicon-bridged xanthene core offers the opportunity for radiofluorination with SiFA isotope exchange to obtain a small-molecule, PET-activatable NIR dye that can be linked to different target vectors. Herein, we demonstrate for the first time the PET-activation of a fluorinated silicon pyronine, belonging to a class of low-molecular-weight fluorescence dyes with a large Stokes shift (up to 129 nm) and solvent-dependent NIR dye properties, with a successful radiochemical conversion of 70%. The non-fluorinated pyronine precursor is easily accessible by a three-step sequence from commercially starting material with a 12% overall yield. Moreover, a library of seven unusually functionalized (by approximately 15 nm), red-shifted silicon rhodamines were synthesized in three- to four-step sequences and the optical properties of the novel dyes were characterized. It was also shown that the synthesized silicon rhodamine dyes can be easily conjugated by amide bond formation or ‘click-reaction’ approaches.

## 1. Introduction

**Figure 1:**
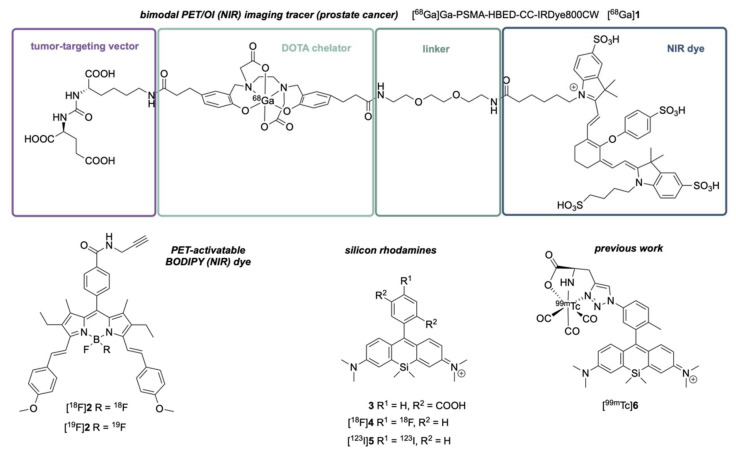
The PET-activatable moiety (DOTA chelator to complex gallium-68) and the NIR dye (IRDye800CW) are separated in the PSMA-targeting bimodal (PET/OI) imaging tracer **1**. In contrast, the bis-styrene BODIPY dye **2** can be PET-activated itself and possesses a significantly reduced molecular weight as a bimodal imaging tracer conjugate. SiR dyes like **3** are NIR dyes with an even lower molecular weight than **2**. However, PET- or SPECT-activation of **4**, **5**, and **6** could not be achieved via an isotope exchange reaction.

Over the past decades, novel molecular imaging methodologies have revolutionized the way to detect malignancies on macroscopic and microscopic levels. In particular, new specific PET (positron emission tomography) tracers, like PSMA- (prostate-specific membrane antigen) or somatostatin-receptor-targeting compounds, have found their way into the clinical routine and impacted clinical treatment direction. For solid cancers, like prostate cancer or neuroendocrine tumors, hybrid PET/CT or PET/MRI (CT: computed tomography, MRI: magnetic resonance imaging) scans can provide valuable information for subsequent therapy (such as external beam radiation therapy [1], radioligand therapy, and/or surgical removal of the tumor [2]). For therapy planning, the information from the static three-dimensional PET images cannot be directly transferred into the surgical setting: malignant tissue is sometimes indistinguishable from healthy tissue, and small anatomical structures, like lymph nodes, are difficult to identify. As surgeons aim for a margin-negative resection, there is a need for a reasonable balance between tissue-sparring surgery and cancer-free margins. To this end, intraoperative fluorescence-guided surgery with biocompatible near-infrared (NIR) dyes can enable surgeons to perform complete and margin-negative removal of primary cancer and metastases while sparing healthy tissue (such as non-affected nerves) [3]. Thus, uniting the modalities of optical imaging (OI) and PET is a logical consequence of combining the advantages of both [4]: while the PET signal offers excellent tissue penetration, OI provides a high spatial resolution without ionizing radiation. To design tumor-specific and bimodal (PET/OI) tracers, a target-binding vector needs to be ligated with a NIR-I or NIR-II dye (e.g., cyanine dyes such as the NIR-II dye indocyanine green (ICG)) and a PET-activatable building block (e.g., DOTA (2,2′,2″,2‴-(1,4,7,10-tetraazacyclododecane-1,4,7,10-tetrayl)tetraacetic acid) chelator, complexing PET radionuclides like gallium-68). Using this concept, the PSMA-targeting hybrid PET/OI (NIR) tracer **1** (Figure 1) was synthesized and translated into an animal model [5] (another derivate (PSMA-914) was recently translated into the human setting [6]). The conjugation of those two building blocks leads to a higher molecular weight (often resulting in slower clearance) and altered lipophilicity, which can result in a lower target affinity and off-target binding [7,8,9,10]. To reduce the molecular weight as well as to minimize undesired effects such as unspecific binding, NIR dyes were developed that can be functionalized with PET radionuclides (like fluorine-18): NIR BODIPY (boron-dipyrromethene scaffold) dyes exploit the high bond energy between boron and fluoride and enable the generation of a PET-activatable NIR dye [11]. While BODIPY dyes require an expansion of the aromatic structure to shift the absorption and emission wavelengths into the NIR region (>650 nm, such as the bis-styrene derivative **2**, λ_abs._ = 654 nm, λ_em._ = 681 nm) [12], silicon rhodamines (SiRs) (such as **3**) are by design NIR dyes, as their silicon center in the conjugated aromatic backbone innately red-shifts their spectral properties [13]. For red-shifted BODIPY dyes, the aromatic expansion comes at the expense of higher lipophilicity and reduced water solubility. Recently, we demonstrated the successful PET- (with fluorine-18) and SPECT-activation (with iodine-123) (SPECT: single-photon emission computed tomography) of a boronic-acid-functionalized SiR precursor, resulting in the radiolabeled NIR dyes [^18^F]**4** (λ_abs._ = 649 nm, λ_em._ = 665 nm) and [^123^I]**5** (λ_abs._ = 648 nm, λ_em._ = 667 nm) with lower masses compared to other NIR BODIPY-derived dyes [14]. Other work proved that triazole-fused SiR is capable of chelating technetium-99m, resulting in a SPECT-activable NIR dye [^99m^Tc]**6** [15]. Nevertheless, PET-activation of BODIPY structures by isotope exchange (IE) of fluorine-19 with fluorine-18 leads to a structurally equivalent product with the same pharmacological behavior, rendering any elaborated separation of the chemically distinct precursor [^19^F]**2** from the PET tracer [^18^F]**2** (in principle) unnecessary. The concept of IE was also harnessed in the SiFA-IE (silicon fluorine acceptor isotope exchange) methodology [16,17,18,19,20] to readily PET-activate a bis(*tert*-butyl)silyl-F or a bis(*diiso*-butyl)silyl-F moiety with a fluorine-18/cryptand reaction mixture. The methodology offers high radiochemical yields within a short reaction time as well as mild reaction conditions that are suitable for compounds sensitive to heat, metal catalysts or extreme pH values. The technology was well established for (pre)clinical imaging of targets such as the cholecystokinin-2 receptor (CCK-2R) [21], the somatostatin rector [22,23], the glucagon-like peptide-1 receptor (GLP-1R), PSMA [24], and for bi-specific imaging of the integrin α_v_β_3_ and the melanocortin 1 (MC1) receptor [25]. The silyl-fluoride moiety can thereby be incorporated in different prosthetic groups like heteroarylic ‘HetSiFAs’ [26], bivalent scaffolds suitable for the design of dimeric radioligands [27], and tetrazine building blocks suitable for ligation by inverse-electron demand Diels-Alder reactions [28]. As the radiofluorination of the aforementioned boronic acid functionalized SiR precursor (yielding [^18^F]**4**) requires a high temperature of 120 °C and stochiometric amounts of copper catalyst, we recognized the potential of SiFA-IE for its extension on PET-activable SiR dyes conjugated to heat- and metal-sensitive vectors.

**Figure 2:**
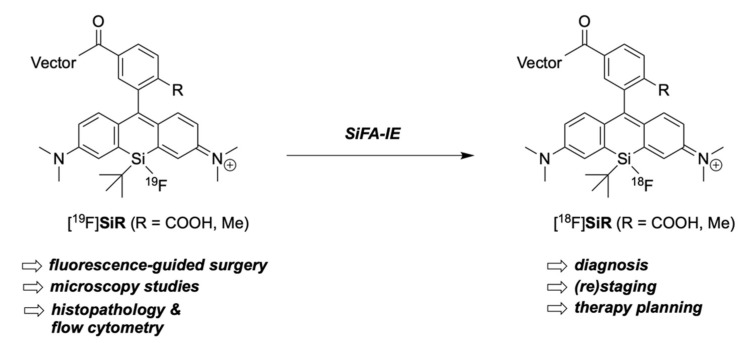
The non-radioactive bimodal imaging tracer [^19^F]**SiR** can be clinically applied for fluorescence-guided surgery or as a probe for (preclinical) microscopy studies. The PET-activated and radioactive compound [^18^F]**SiR** can be clinically utilized for diagnosis, (re)staging, and therapy planning (surgical intervention, radiotherapy, and targeted radioligand therapy). Furthermore, it allows us to correlate tumor uptake in PET with immunohistochemistry or FACS (fluorescence-activated cell sorting) results.

The aim of this research was to examine if the silicon center of a SiR can be utilized for radiolabeling via the SiFA-IE strategy to result in a low-molecular-weight PET-activatable NIR dye for bimodal imaging that can be linked to different (tumor-specific) targeting vectors (e.g., PSMA-binding motif) (Figure 2). The usage of fluorine-18 as PET radionuclide promises high-resolution images for staging or therapy planning (e.g., surgery) with PET/CT or PET/MRI after administration of the radioactive [^18^F]**SiR**. For fluorescence-guided surgery, the non-radioactive surrogate [^19^F]**SiR** can be administered, as it shows the same pharmacodynamic and -kinetic profile (nota bene: pharmacokinetic properties are affected by the dosage; the dosage would be greater for fluorescence-guided surgery). In principle, the strategy allows both the radioactive and non-radioactive compounds to be clinically approved in one and the same way since their structures are identical (nota bene: surgical applications will require higher dosages compared to microdosing for PET image acquisition; therefore, requirements for the assessment of non-clinical data (such as toxicity data) might be different).

## 2. Results

### 2.1. Synthesis of the Silicon Xanthone ***8***

To harness the silicon in the SiR scaffold for the covalent bonding of fluorine, the other methyl group was replaced with a *tert*-butyl group to kinetically stabilize the silicon-fluorine bond, according to published work on precursors for SiFA-IE [18]. For fluorination, a silyl methoxy ether was envisioned as a suitable functional group. By now, no SiR dyes (or precursors suitable for SiR synthesis) with alkoxy substituents or with a *tert*-butyl-substituent have been published, and most of the published SiR dyes show a symmetrical (mainly dimethyl) substitution pattern. Recently, Miller et al. achieved the synthesis of a library of Si-bridge-modified SiRs with, in part, two different substituents such as aromatics, alkyl chains, and linkers suitable for ligation (e.g., HaloTag, halogen propyl, azidopropyl, NHS ester) [29].

**Figure 3:**
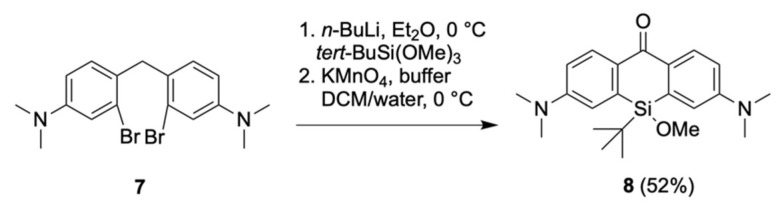
Si-xanthone **8** can be readily synthesized from bis-aniline **7**.

**Figure 6:**
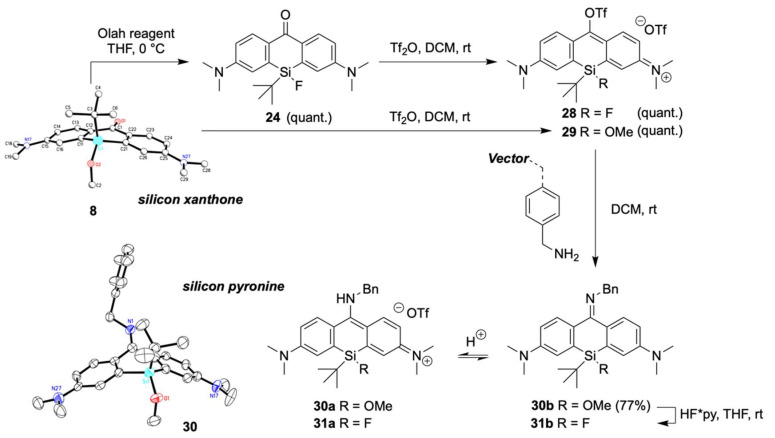
The silicon-substituted pyronines **30** and **31** could be obtained over two reaction sequences. Benzylamine was used as a model amine representative for any amine functionality of various targeting vectors, which can be ligated with the pyronine tag. In polar solvents and at low pH, the pyronines can adopt a zwitterionic structure (**30a** and **31a**), which exhibits NIR properties.

The methoxy *tert*-butyl-substituted Si-xanthone **8** was synthesized as a key intermediate over a two-step sequence from bis-aniline **7**, which was derived from the known condensation of bromo-aniline with formaldehyde (Figure 3) [30]. Lithiation of **7** and subsequent reaction with *tert*-butyl-Si(OMe)_3_ led to the silicon heterocycle, and permanganate oxidation with an optimized buffer [31] furnished Si-xanthone **8** in 52% yield over two steps on a multi-gram scale. Additionally, crystallography confirmed the identity of Si-xanthone **8** (see crystal structure in Figure 6).

### 2.2. Functionalization of Silicon Xanthone ***8*** to Silicon Rhodamines

**Figure 4:**
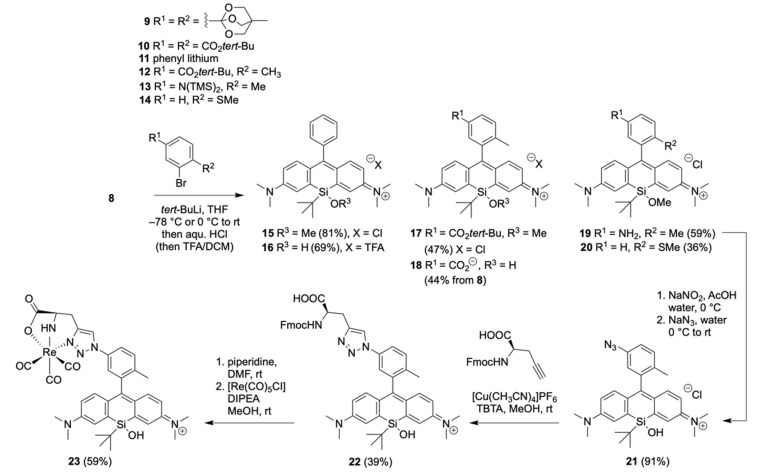
Synthesis of SiRs containing a (*tert*-butyl)MeOSi-motif. Amine **19** can be converted to azide **21** via diazotization. Copper-catalyzed click chemistry leads to triazole **22**, which can complex metals such as rhenium or (SPECT-active) technetium. TBTA—tris(benzyltriazolylmethyl)amine.

While we could previously show that xanthones like **8** can be triflated and used for subsequent Suzuki-Miyaura couplings to obtain SiR dyes [32], in the present study, we opted for a SiR synthesis via the addition of an aryl lithium species, as the cross-coupling did not tolerate the introduction of a terephthalic acid moiety. Therefore, we assessed the lithiated and fully protected terephthalic building blocks **9** and **10** for the addition to the Si-xanthone **8** (Figure 4). While neither of them reacted, quick and full conversion was observed with the phenyl lithium (**11**), resulting in the *tert*-butyl/methoxy silicon-bridged dye **15** in 81% yield. Depending on the work-up, the methyl group of the ether was lost, and the corresponding silanol dye **16** was obtained in 69% yield. As substituents on the phenyl ring are required for further ligation with a biovector as well as for conformational restriction (prevent rotation around the xanthene-phenyl-axis to achieve a high quantum yield), other aromatic building blocks were screened. Lithiated and protected *para*-toluic acid (**12**) showed not only good reactivity but also contained a reasonably sized substituent to provide conformational restriction. In this way, the SiOMe-substituted ester **17** was obtained in a 47% yield. Subsequent deprotection resulted in the silanol dye **18**, which contained a free acid functionality for ligation with a vector of interest. As a methyl substituent might represent the upper size limit, smaller substituents like OMe or SMe (as in **20**) seemed to be tolerated well in the addition reaction. Also, the ester functionality could be exchanged with other functional groups capable of ligation, as exemplified with the azide **21**, which was synthesized from the bis(TMS)-protected amine **19** in 91% yield and can be used for bioorthogonal ligation via ‘click-chemistry’. In this way, we were able to show that **21** underwent cycloaddition to form triazole **22**, which could be further converted to the rhenium complex **23** (note: molecules **22** and **23** were only characterized by HR-MS, as purification by HPLC was not performed, and yields are thus only indicative; syntheses were performed according to [15]). With SiRs possessing a Si(Me)_2_-substitution pattern, we could show that rhenium complexes of SiRs resemble non-radioactive surrogates of the analog SPECT-active ^99m^Tc-complexes [15] (see also Figure 1).

To avoid the formation of rotamers by the introduction of a symmetrical phenyl part (as observed for **17** or **18**), we attempted to determine if lithiated mesitylenic acid could be introduced. However, no conversion was observed with this building block.

To show the capability for follow-up reactions, acid **18** was converted to a TFP ester that readily underwent amide formation with a PSMA-targeting motif (Glu-Ureido-Lys-NHCO-Ph-CH_2_NH_2_).

### 2.3. Fluorination of Silicon Rhodamines

**Figure 5:**
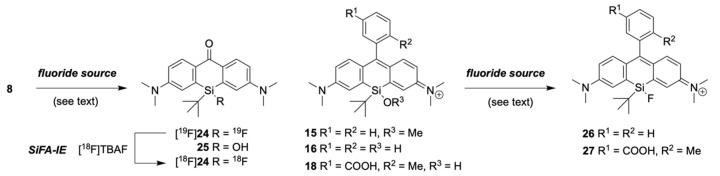
By using different nucleophilic fluoride sources (see text), it was possible to convert model compound **8** into the corresponding silyl fluoride **24**, which underwent SiFA-IE. The fluorination of SiR **16** to **26** was accomplished on a small scale and monitored by ^19^F-NMR, but **26** underwent rapid hydrolysis under aqueous conditions.

As the application of silyl fluorides is limited, knowledge about their reactivity is scarce. Therefore, we first explored the fluorination with non-radioactive fluorine-19 on Si-xanthone **8** as a model compound (Figure 5). Fluoride sources like KF/Kryptofix^®^ 2.2.2 and the Olah reagent led up to the quantitative formation of the fluorinated Si-xanthone **24** (29% yield with aq. hydrogen fluoride), while TBAF solely led to the formation of the silanol **25** in 77% yield, although clear Si-F formation could be detected by ^19^F-NMR (typical Si-F shift at around −187 ppm [33]) directly after the addition of TBAF. Nevertheless, silanol **25** could be converted to the corresponding fluoride by either KF/Kryptofix^®^ 2.2.2 or the Olah reagent. Although TBAF is a common reagent for the cleavage of silyl-protecting groups, TBAF is not a typical reagent for the synthesis of Si-F species. Interestingly, Si-xanthone **24** could be readily radiolabeled under standard SiFA-IE conditions with an azeotropic dried complex of [^18^F]F^−^/Kryptofix^®^ 2.2.2/K^+^*,* yielding [^18^F]**24** (identity and stability over several hours were confirmed by (radio-)HPLC with [^19^F]**24** as non-radioactive reference standard). Moreover, **24** was stable in water, and almost no hydrolysis was observed after 40 min in saturated bicarbonate solution (t_1/2_ of [^19^F]**24** in DIPEA/MeCN: >4 h; in 0.05 N HCl: 70 min, all by HPLC).

Next, the syntheses of the dyes [^19^F]**26** and [^19^F]**27** were attempted. Neither the use of silyl methyl ether **15**, nor of the silanol dyes **16** or **18** led to an identifiable product with KF/Kryptofix^®^ 2.2.2, aq. HF, DAST, BF_3_*OEt_2_, TBAF, Selectfluor^®^, or the Olah reagent. As the SiR dyes are salts, we assumed that the nucleophilicity of the counter ions (usually from an excess source of chloride due to work-up with hydrogen chloride) would interact with the intermediately formed silyl fluoride. Dissatisfyingly, with triflate or trifluoroacetate acid anion as a non-nucleophilic counterion, **26** or **27** could not be identified in the reaction mixture under the conditions mentioned before. Finally, the methoxy silanol ether **15** underwent fluorination to **26** with an aqueous solution of hexafluorosilicic acid in acetonitrile/THF at room temperature as proved with ^19^F NMR (typical Si-F shift at −185 ppm), HR-MS, and HPLC. However, isolation could not be achieved, as **26** underwent rapid hydrolysis to silanol **16** during work-up and/or during purification with SiO_2_ (water-free conditions with (NH_4_)_2_SiF_6_ also resulted in hydrolysis). Although this result seemed promising at first glance, hexafluorosilicate anions might be problematic for the subsequent isotopic exchange reaction with fluorine-18, as they could drastically decrease the molar activity and the radiochemical yield. This reasoning is based on the possibility that both the silicate counterion as well as the SiR could undergo an isotopic exchange reaction. Other substrates, such as **19**, **21** and **22**, were examined in the fluorination reaction with hexafluorosilicic acid, but only **22** showed conversion to its corresponding fluoride as judged by HPLC-MS (no work-up was attempted).

Other strategies to synthesize fluorinated SiR dyes like **26** (i.e., the addition of phenyl lithium (**11**) to the fluorinated xanthone **24**) failed due to the incompatibility of the Si-F bond with strong nucleophiles (such as organolithium compounds) and/or strong bases.

### 2.4. Functionalization of Xanthone ***8*** and ***24*** to Silicon Pyronines

Exploiting the conditions published by Hell and coworkers, xanthone **8** was converted to the triflated pyronine **29** by the addition of triflic anhydride (Figure 6) [34]. Due to the high reactivity of the resulting pyronine with amines, the triflate **29** was seen as a good reactant for a tag-like ligation with a specific vector (a similar tag concept with O-pyronines was employed by Hymel and coworkers [35]). In this way, **29** reacted quickly with benzylamine and complete conversion was indicated by a powerful color change from deep blue to bright orange. The silicon-substituted pyronine **30** was obtained in 77% yield (fluorescent properties can be found in Section 2.6). Interestingly, X-ray analysis of **30** revealed a bent structure of the tricyclic core, while Si-xanthone **8** showed an almost flat geometry (Figure 6).

In addition, other nucleophiles were screened for the reaction with Si-xanthone **8** in DCM or THF, but no corresponding pyronine could be obtained with benzyl mercaptan, phenyl sulfonamide, methyl sulfonamide, or hydroxylamine.

### 2.5. Fluorination of Silicon Pyronines

**Figure 7:**
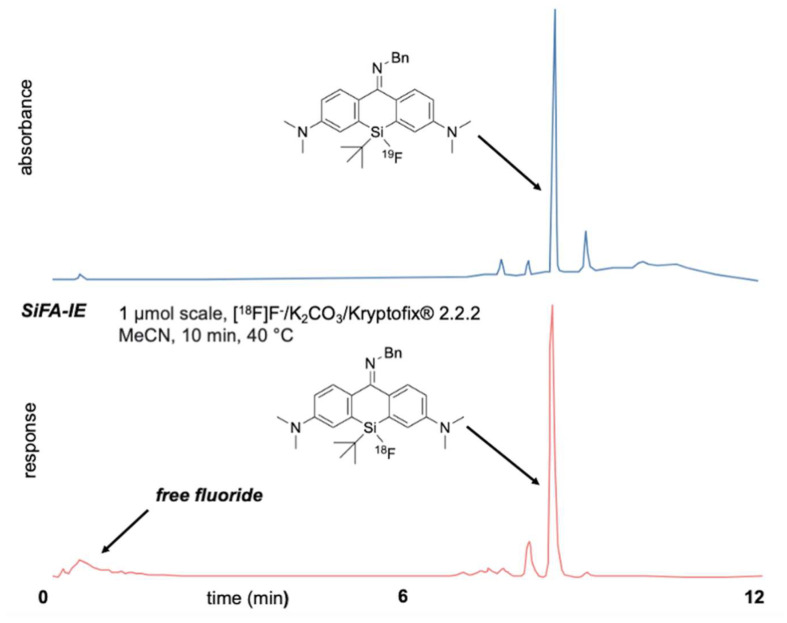
Successful SiFA-IE of [^19^F]**31** with azeotropic dried [^18^F]KF: the radiolabeled pyronine [^18^F]**31** could be clearly detected by (radio-)HPLC (lower graph), the non-radioactive compound (crude, due to instability during chromatography) was used as a reference (upper graph) (see Supplementary Materials for details). The side-products were not characterized.

With pyronine **30** at hand, fluorination to **31** could be performed with the Olah reagent, aqueous HF, or DAST. However, no formation of **31** was observed by using TBAF, KF/Kryptofix^®^ 2.2.2 or hexafluorosilicic acid (Figure 6). As an alternative route, the fluorinated xanthone **24** could be readily transformed into the fluorinated pyronine **28** with the addition of triflic anhydride, followed by the addition of benzylamine, leading to the formation of pyronine **31**. Regardless of the synthetic route, purification (with silica gel, C_18_-functionalized silica, or deactivated neutral aluminum oxide (Alox) purification) afforded the pyronine silanol. Nevertheless, the crude product was already obtained in sufficient purity for radiolabeling. A small sample of [^19^F]**31** was treated with azeotropic dried [^18^F]KF in MeCN for 10 min at 40 °C, and the formation of [^18^F]**31** could be clearly detected by (radio-)HPLC (Figure 7). The radiofluorinated silicon-containing pyronine dye [^18^F]**31** was labeled with a radiochemical conversion of 70%. While the dye showed relatively good water stability (t_1/2_: 3 h), the addition of [^18^F]**31** into DMSO/serum albumin/PBS resulted in rapid decomposition (t_1/2_: 5 min). The determined logP = 0.73 of [^18^F]**31** indicated that this compound is rather hydrophilic.

### 2.6. Optical Properties of Silicon Rhodamines and Pyronines

For the optical characterization of the new SiRs and pyronines, the absorption and emission spectra, the quantum yields, and the extinction coefficients were measured (Table 1 and Supplementary Materials). The spectra of SiRs **15**, **16** and **18** exhibit absorption and fluorescence behavior above 650 nm, which is a basic requirement for in vivo imaging (wavelength frame: 650–900 nm). The rather low quantum yields of the 2′-unsubstituted SiRs **16** (14%) and **15** (28%) can be explained by the lack of a substituent (such as the methyl group in **18** or **32** or the SMe-substituent in **20**) in the 2′-position, which would have prevented the rotation of the phenyl ring in the phenyl-silicone xanthene axis. In contrast, the quantum yield of the conjugatable dye **18** reached 50%, arguably due to the introduction of the methyl group in position 2′, while the spectral properties stayed similar to those of **15** and **16**. Compared to the already known non-PET-activatable dimethyl-substituted SiR **32**, a red shift of approximately 15 nm was found for **15** and **16**, which was probably caused by the methoxy/hydroxy substituents on the silicon atom interacting additionally with the conjugated system. This is the first time that the effect of an alkyl/hydroxy(methyl) silicon substituent pattern on the fluorescence behavior could be studied. Dye **33** exhibited similar absorbance and emission maxima like **15** or **16** and is the only published example of a SiR with two hydroxyl groups installed at the silicon center [36].

For all examples, the silanol or silanol ether motifs led to a red-shift of up to 20 nm (for Re-complexes, **23** vs. **37**), and the extinction coefficient was reduced for the Si-OR substituted SiRs compared to their Si(Me)_2_ analogs. However, no clear trend was observed for quantum yields. In addition, no relevant influence of solvents was detected (except for one example: the quantum yield of **21** increased dramatically in MeCN).

The fluorescence spectrum of pyronine **30** (see Figure S1) shows three absorption maxima (322 nm, 390 nm, 478 nm), with the absorption being strongest at 478 nm. Excitation at the individual absorption maxima always resulted in emission at approx. 607 nm. In contrast, its fluorinated counterpart, pyronine **31**, exhibits a dominant absorption band with a maximum of 325 nm and two main emission bands, with the emission band at 599 nm being weaker than that at 461 nm.

Characteristic of the pyronine dye class [34,37], the Stokes shifts of the pyronines **30**, **31,** and **38** are remarkably high, measuring 129 nm (λ**_30_**_,abs._ = 478 nm, λ**_30_**_,em._ = 607 nm), 119 nm (λ**_31_**_,abs._ = 480 nm, λ**_31_**_,em._ = 599 nm), and 145 nm (λ**_38_**_,abs._ = 462 nm, λ**_38_**_,em._ = 607 nm), respectively. As reported [34,37], the fluorescence behavior of pyronines depends on solvents and pH: protic solvents and low pH shift the equilibrium towards the polar, cationic structure (see equilibrium of **30** in Figure 6). In this case, the cationic isomer does not show a large Stokes shift, but both absorption and emission bands shift into the NIR region. The solvent and pH effects on the NIR fluorescence properties of **30** were not recorded, as previous publications on pyronines (e.g., **38**) suggest similar solvent-dependent optical properties [34,37]. In summary, the spectral properties of **31** are more complex than that of the non-fluorinated analog compound **30**, indicating simpler photophysics of the latter.

## 3. Discussion

This research aimed at evaluating the accessibility of the silicon center of a SiR for radiofluorination via SiFA-IE to generate a low-molecular-weight PET-activatable NIR dye for bimodal imaging that can be linked to different (tumor-specific) target vectors. In the first step towards this goal, we established a multi-gram scale synthesis of silicon xanthone **8**. With **8** at hand, a library of SiRs and two pyronines was successfully synthesized. The SiRs were decorated with a distinct pattern at the silicon center, which is required for the late-stage fluorination step. In contrast to the syntheses of classical Si(Me)_2_-bridged SiRs, the size of the 2′-substituent at the aryl lithium compound was a limiting factor for the reactivity between silicon xanthone **8** and the lithiated aryl. While a 2′-methyl or 2′-thiomethyl ether was well tolerated as a substituent, all protected carboxylic acids were not. Although the installation of a carboxylate would have been beneficial for polarity, the solubilities of all synthesized SiRs were satisfactory.

Introducing an oxygen substituent at the silicon center resulted in a remarkable red-shift of up to 20 nm compared to traditional Si(alkyl)_2_-substituted SiRs. Although such substituted silicon rhodamines did not form water-stable silyl fluorides, we expect this finding to help further fine-tune the optical properties of this dye family towards the NIR region. Furthermore, we hypothesize that the installation of the *tert*-butyl at the silicon center is beneficial beyond kinetic stabilization of the silicon-fluorine bond, as its bulky nature may prevent the aggregate formation of the fluorophores by suppressing H-stacking of the dye molecules. In general, aggregate formation is an undesired phenomenon and is known to quench the fluorescence of SiRs [39,40,41]. Conclusively, although (stable) PET-activation was not achieved with the SiRs, the dyes’ photophysical properties meet the requirements of various different OI methodologies (e.g., STED (STimulated Emission Depletion) nanoscopy [42,43] or fluorescence-guided medical imaging).

As the fluorinated pyronine **31** could be successfully isolated, we wondered why the silyl fluoride bond in fluorinated SiRs seemed to be more susceptible to hydrolysis and assumed that the antibonding orbital of the Si-F bond might be more readily available for the attack of water if the silicon was part of the aromatic xanthene tricycle (and rather less available, if part of the pyronine core). To prove this hypothesis, the stability of the fluorinated triflate **28** was examined by NMR. We found that **28** was stable over several hours, and even after the addition of water, signals of silanol **25** increased only very slowly. Therefore, the reason for the instability of the fluorinated SiRs remains unclear. As a general strategy to increase the water stability of the SiRs and pyronines, replacing the *tert*-butyl group with a sterically more hindered group (e.g., adamantyl group) would certainly stabilize the Si-F bond, and the installation of additional alkyl groups at the rhodamine core could provide further kinetic stabilization. It must be noted that usual building blocks for SiFA-IE radiolabeling feature two *tert*-butyl, two *iso*-butyl, or two *iso*-propyl groups at the silicon center, but that also (radioactive) Ph_2_(*tert*-butyl)Si-F proved to be stable in vitro in human serum [16].

While a plethora of SiRs have been published, the family of silicon pyronines is still comparatively small. As for SiRs, no silicon pyronines bearing a silanol ether motif have been reported to date. We demonstrated that this kind of silicon center could be installed in pyronine **30**, and that the methoxy group in **30** could be successfully substituted with fluoride. As the pyronine dye is formed during conjugation with an amine function (of a model vector molecule), the synthesis is step-economic. Furthermore, the color change from deep blue (triflates **28** or **29**) to bright orange clearly and conveniently indicates the reaction end. The absorption and fluorescence spectra of both **30** and **31** in MeCN showed, as expected, a large Stokes shift. It is known that pyronines exhibit NIR dye properties in protic or acidic media as polar conditions shift the equilibrium to their charged structures like **30a** or **31a** [34,37].

Although **31** could be synthesized in sufficient purity after a short work-up, silanol formation was observed during purification with silica gel, C_18_-modified silica gel, or deactivated neutral Alox. We reasoned that the vast number of Si-OH or Al-OH functionalities led to silanol formation, as these functionalities can accept the fluoride from **31** (the transit of fluoride between two silicon centers should be thermodynamically energy-neutral). Nevertheless, even without purification, sufficient purity of **31** was achieved, and [^19^F]**31** was successfully radiolabeled under SiFA-IE conditions with 70% radiochemical conversion, affording [^18^F]**31**.

Still, susceptibility to hydrolysis needs to be addressed in an improved second generation of SiR-based PET-activatable dyes. To this end, basic adaptions include the replacement of the *tert*-butyl-group with a bulkier substituent and the decoration of the pyronine core with bulky groups. Furthermore, the polar isoform of pyronines, exhibiting NIR dye properties, should be stabilized, for example, by substitution of the imine group.

Taken together, we here introduce for the first time the fluorinated silicon pyronine **31** as a novel low-molecular-weight tracer core with a broad Stokes shift and solvent-dependent NIR dye properties, readily PET-activatable and vector-conjugatable. We successfully demonstrated its radioactivation by SiFA-IE with a satisfying radiochemical conversion. Moreover, we present a library of unusual functionalized, red-shifted SiRs that can be further functionalized by amide bond formation or ‘click-chemistry’.

## 4. Materials and Methods

General methods, synthetic procedures, fluorescence spectra, and analytical data can be found in the Supplementary Materials.

## Figures and Tables

**Table 1:** Optical data of SiRs and pyronines.

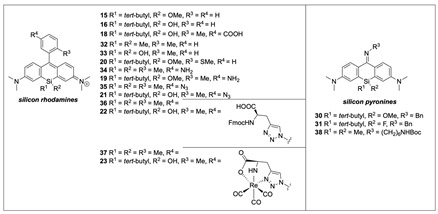					
**Compound**	**Solvent**	**λ_abs_. (nm)**	**λ_em_. (nm)**	**ϕ_fl_**	**ε_max_ (M^−1^ cm^−1^)**
**15**	MeCN	663	681	0.28 ^1^	109,410
**16**	PBS ^2^	659	685	0.14 ^1^	-
**18**	MeCN	664	684	0.50 ^1^	-
**32** ^4^	PBS ^2^	646	660	0.31 ^4^	-
**33** ^5^	PBS ^6^	663	681	0.43	105,000
**20**	MeCN	672	696	0.55 ^1^	89,124
**34** ^7^	MeOH	653	-	-	91,900
	PBS ^2^	651	-	-	77,300
**19**	MeOH	664	679	0.074 ^1^	67,750
	H_2_O ^3^	665	680	0.052 ^1^	51,600
	PBS ^2^	663	678	0.026 ^1^	61,900
	MeCN	665	687	0.010 ^1^	120,000
**35** ^7^	MeOH	651	670	0.175 ^1^	156,500
	H_2_O ^3^	651	670	0.103 ^1^	123,700
	PBS ^2^	651	671	0.116 ^1^	99,000
**21**	MeOH	663	680	0.076 ^1^	73,900
	H_2_O ^3^	663	677	0.054 ^1^	77,200
	MeCN	664	685	0.450 ^1^	98,000
**36** ^7^	MeOH	655	672	0.130 ^1^	79,900
	H_2_O ^3^	653	671	0.098 ^1^	73,890
	PBS ^2^	655	672	0.127 ^1^	79,900
**22** *	H_2_O ^3^	671 *	693 *	- *	- *
**37** ^7^	MeOH	654	672	0.135 ^1^	63,900
	H_2_O ^3^	654	674	0.104 ^1^	22,100
	PBS ^2^	654	669	0.090 ^1^	39,100
**23** *	PBS ^2^	671 *	693 *	- *	- *
**30**	MeCN	323 390 478	607 ^8^	n.d.	n.d.
**31**	MeCN	323 (strong) 393 480	457 ^9^ (weak) 461 ^10^ (strong) 599 ^11^ (medium)	n.d.	n.d.
**38** ^12^	MeCN	317 462	607 ^13^	0.58	21,000

^1^ For the determination of the quantum yields, Nile blue in ethanol was used as a reference dye. ^2^ Values were determined at pH 7.4. ^3^ Samples contained 5% ethanol as an additive. ^4^ Synthesis and optical data reported by Koide et al. [38]. For the determination of the fluorescence quantum yield, cresyl violet in methanol was used as a fluorescence standard. ^5^ Synthesis and optical data were reported by Zhou et al. [36]. ^6^ Values were determined at pH 7.4 with 0.1% DMSO. ^7^ Synthesis reported by Kanagasundaram et al. [15]. ^8^ Strongest emission by excitation at 480 nm, intermediate emission by excitation at 390 nm, and weakest emission by excitation at 325 nm. ^9^ Excitation at 325 nm. ^10^ Excitation at 390 nm. ^11^ Excitation at 480 nm. ^12^ Synthesis and optical data reported in [34]. ^13^ Excitation at 470 nm. * Compounds were not subjected to HPLC separation; therefore, ϕ_fl_ and ε_max_ would be falsified. n.d. not determined.

## Data Availability

General methods, synthetic procedures, fluorescence spectra, and analytical data can be found in the Supplementary Materials.

## Supplementary Materials

The following supporting information can be downloaded at: https://www.mdpi.com/article/10.3390/ph16030401/s1, Figure S1: Normalized absorption (abs) and emission (fluo) spectra of SiR **15** (max. abs at 663 nm, max. fluo at 681 nm with excitation at 640 nm), pyronine **30** (abs at 323 nm/390 nm/478 nm, max. fluo at 607 nm with excitation at 480 nm) and pyronine **31** (abs at 323 nm/393 nm/480 nm, max. fluo at 599 nm with excitation at 480 nm) in MeCN; Figure S2: Relative measurement of the quantum yield of SiR **15** in MeCN (n = 1.34) using Nile blue as reference standard in 5% (*v*/*v*) 0.1 M HCl in EtOH ($n_{ref}$ = 1.36); Table S1: Fit Parameter for the relative measurement of the quantum yield of SiR **15** in MeCN using Nile blue as reference standard. General methods, synthetic procedures, fluorescence spectra, and analytical data, CCDC 2232514 (**8**) and 2232515 (**30**) contain supplementary crystallographic data for this paper. These data can be obtained free of charge from The Cambridge Crystallographic Data Centre via www.ccdc.cam.ac.uk/data_request/cif. Refs [30,44,45,46,47,48,49,50] are cited in the supplementary material.

## Sample Availability

Some samples may be available on request.
