# Supplementary material for: Efforts toward PET-Activatable Red-Shifted Silicon Rhodamines and Silicon Pyronine Dyes
Source: Pharmaceuticals (Basel). 2023 Mar 7;16(3):401. doi: 10.3390/ph16030401 (PMC10053042; doi:10.3390/ph16030401)
Supplement: Supplementary file 1 [file pharmaceuticals-16-00401-s001.zip › pharmaceuticals-2180269-supplementary.pdf]

Supporting Information

–

Efforts toward PET-activatable red-shifted silicon rhodamines and silicon pyronine dyes

Table of contents

General remarks 2

Kryptofix® 2.2.2 2

Synthesis of silicon xanthone 8 5

Syntheses of bromo-aryl building blocks 9, 10, 12, and 13 8

Syntheses of the silicon rhodamines 15, 16, 17, 18, 19, 20, and 21 12

Syntheses of silyl fluorides 24, 28, and of silanol 25 19

Synthesis of silicon pyronines 30, 31, and [^{19}F]31 21

Fluorination of silicon pyronine 30 to 31 23

Radiolabelling of 31 via SiFA-IE 24

Optical spectroscopy 26

4.1 Absorption and emission 26

4.2 Fluorescence quantum yield 26

General remarks

If not otherwise described, reactions requiring exclusion of oxygen and moisture were carried out in heat-gun flasks dried under argon gas atmosphere using the Schlenk-technique.

All **chemicals** and **solvents** were used as received without other purification. Deuterated solvents for NMR were purchased from Deutero GmbH.

Chemical	Supplier (stock-keeping unit)
2-bromoterephthalic acid	8414670005, Sigma Aldrich
2-bromothioanisole	Sigma Aldrich, 260851-5G
2,6-lutidine	Thermo Fisher, Sigma Aldrich
3-bromo-4-methyl-aniline	Sigma Aldrich, 360619-5G
3-bromo-4-methylbenzoic acid	Sigma Aldrich
3-bromo-*N,N*-dimethylaniline	VWR
4-DMAP	Acros Organics
benzylamine	Sigma Aldrich, 185701-5G
boron trifluoride diethyl etherate	Sigma Aldrich, 175501-4X25ML
n-BuLi (2.5 M in *n*-hexane)	Sigma Aldrich, 230707-4X25ML
tert-BuLi (1.7 M in pentane)	Sigma Aldrich, 186198-4X25ML
tert-butyl-trimethoxysilane	Abcr GmbH, no longer in catalogue
formaldehyde (37 wt% in water, stab. with 5-15% methanol)	Fisher Scientific
K_2CO_3 (anhydrous, for SiFA-IE)	Sigma Aldrich, 590681-5G
Kryptofix® 2.2.2	Sigma Aldrich, 8106470001
LiHMDS (1.0 M in THF)	Sigma Aldrich, 225770-4X25ML
3-methyl-3-oxetanemethanol	Sigma Aldrich, 277681-10G
Olah reagent	Sigma Aldrich, 184225

phenyllithium (1.9 M in dibutyl ether)	Sigma Aldrich, 593230-100ML
sodium azide	Sigma Aldrich, S2002-5G
sodium nitrite	Sigma Aldrich, 237213-5G
TBAF (1.0 M in THF)	Sigma Aldrich, 216143-100ML
triflic anhydride solution (1.0 M in DCM)	Sigma Aldrich, 704083-25ML
trimethylsilyl chloride	Sigma Aldrich, 92361-100ML

Commonly used chemicals (such as sodium chloride, thionyl chloride, Boc anhydride, acetic acid, 37% hydrochloric acid, tetrabutylammonium bisulfate, potassium permanganate, pyridine, triethyl amine, TFA), drying agents (such as sodium sulfate, magnesium sulfate) or solvents for extraction were used from various suppliers (Sigma Aldrich, Acros Organics, Abcr, TCI).

Anhydrous solvents (DCM, THF, MeCN, *n*-hexane, diethyl ether) were purchased from Sigma Aldrich as sure-seal bottles^{TM}. Solvents were drawn under an applied flow of argon/nitrogen or a balloon filled with argon/nitrogen.

NMR spectra were recorded at room temperature on a 400 MHz Bruker Avance III spectrometer. Chemical shifts are reported in δ units relative to methanol-d_4 (δ_H = 3.31; δ_C = 49.0) or chloroform-d_3 (δ_H = 7.26; δ_C = 77.36).[1] Analyses followed first order and the following abbreviations were used throughout: s = singlet, d = doublet, t = triplet, dd = doublet of doublet etc., m = multiplet. Coupling constants (J) are given in Hz and refer to H, H-couplings.

Mass spectra (MS) were determined in the organic chemistry department of the University of Heidelberg under the direction of Dr. Jürgen Gross. The ionization method ESI was applied using spectrometer BrukerApexQe hybrid 9.4 T FT-ICR. High-resolution mass spectra (HR-MS) were recorded on a JEOL JMS-700 spectrometer. The molecule fragments are reported in mass to charge (*m/z*) relation.

X-ray crystallography was performed by Dr. Frank Rominger with an X-ray diffractometer Bruker APEX II Quazar with a Mo-Microsource at the Institute for Organic Chemistry at the University of Heidelberg.

[1] G. R. Fulmer, A. J. M. Miller, N. H. Sherden, H. E. Gottlieb, A. Nudelman, B. M. Stoltz, J. E. Bercaw, K. I. Goldberg, *Organometallics* **2010**, *29*, 2176–2179.

Spectroscopic optical characterization was performed with devices from Agilent, model Cary Eclipse Fluorescence Spectrophotometer (Scan Software Version: 1.2(147)) and Cary 5000 UV-VIS-NIR (Scan Version 6.2.0.1588, Instrument Version: 3.00) at the Max Planck Institute for Medical Research Heidelberg, Department of Optical Nanoscopy.

Analytical Thin Layer Chromatography (TLC) was carried out on polygram-DC-plates produced by Machery-Nagel (40 x 80 mm, SIL G/UV_{254}, 0.2 mm layer thickness). Detection was carried out using UV-light (254 nm or 366 nm). TLC on reversed-phase were carried out on alugram-DC-plates produced by Machery-Nagel (40 x 80 mm, RP-18 W/UV_{254}, 0.15 mm layer thickness).

Radio-HPLC was carried out using the following set-up and flow parameters:

Device	Brand	Type
Pump	Thermo Fischer	LPG-3400SD
UV detector	Thermo Fisher	VWD-3400RS
Chromatography Interface	Thermo Fisher	UCI-50
Radioactivity detector	Raytest	Gabi Star
Column	Merck	Chromolith RP-18 Performance 4.6 x 100 mm

Gradient parameter:

A% [MeCN]	B% [H_2O]	ml/min	time in min
10	90	4	0
90	10	4	12

Flash column chromatography was carried out on silica gel (0.032–0.062 mm, produced by Macherey-Nagel) using manual techniques.

Software: For drawing chemical formulas ChemDraw Professional 16.0.1.4 was used. HPLC graphs were digitalized for publication using https://apps.automeris.io/ and graphically processed using https://plot.ly/.

Synthesis of silicon xanthone 8

4,4'-methylene bis(3-bromo-*N,N*-dimethylaniline) (7)

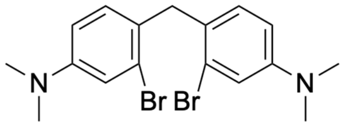

7

In a round bottom flask, 3-bromo-*N,N*-dimethylaniline (16.5 g, 82.3 mmol) was dissolved in 132 mL of acetic acid and 16.5 mL of 37% aqueous formaldehyde solution was added. The reaction mixture was stirred at 60 °C for 30 min. The acetic acid was then removed on the rotary evaporator and the residue was dissolved in ethyl acetate. Carefully (gas evolution!), a saturated aqueous sodium bicarbonate solution was added and the aqueous phase was separated. The aqueous phase was shaken out several times with ethyl acetate and then the combined organic phases were washed first with water, then with brine solution. Subsequently, drying was carried out over sodium sulfate and, after filtering off the desiccant, the solvents were evaporated on the rotary evaporator. The crude product was purified by column chromatography (silica gel, diethyl ether:*n*-hexane 1:9 to 2:8) to give product **7** (10.0 g, 24.3 mmol, 30% yield) as a colorless solid. The analytical data collected are in accordance with the literature (Lukinavicius, G., et al., *A near-infrared fluorophore for live-cell super-resolution microscopy of cellular proteins.* Nat Chem, 2013. **5**(2): p. 132-139).

5-(*tert*-butyl)-3,7-bis(dimethylamino)-5-methoxydibenzo[*b*,*e*]silin-10(5*H*)-one (8)

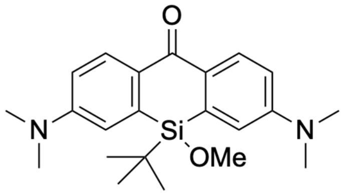

8

In an oven-dried round bottom flask, 4,4'-methylenebis(3-bromo-*N,N*-dimethylaniline) (**7**) (3.60 g, 8.80 mmol, 1.0 equiv.) was dissolved in 85 mL dry diethyl ether under argon. The solution was cooled to 0 °C and subsequently, *n*-BuLi (2.5 M in *n*-hexane, 25.9 mmol, 9.6 mL, 2.9 equiv.) was added at this temperature. The light-yellow mixture was stirred for 1.5 h at 0 °C and *tert*-butyl-trimethoxysilane (1.57 g, 8.80 mmol, 1.0 equiv.) was added dropwise at this

temperature. The reaction was stirred for one day, during which the mixture was warmed to room temperature (note: the yellow reaction mixture turned purple in contact with air). The reaction was quenched by adding diluted brine solution. The aqueous phase was separated and extracted twice with diethyl ether and once with DCM. The combined organic phases were dried over sodium sulfate and after filtering off the desiccant, the solvents were evaporated off on the rotary evaporator. The resulting light brown oil turned blue-green upon prolonged exposure to air. The residue was dissolved in 64 mL of DCM in a large-opening round-bottomed flask (equipped with a powerful stirring bar) and cooled to 0 °C. Potassium permanganate (4.14 g, 26.2 mmol, 3.0 equiv.) previously dissolved in 36 mL (hot) distilled water, 1 M potassium hydroxide solution (8.80 mL, 8.80 mmol, 1.0 equiv.), and tetrabutylammonium bisulfate (600 mg, 1.77 mmol, 0.2 equiv.) were then combined in a separate beaker. Distilled water was added to reach a final volume of 54 mL. The purple solution of the beaker was then added to the round bottom flask under vigorous stirring at 0 °C. This caused the contents of the flask to turn brown. The beaker was rinsed with 36 mL each of DCM and 36 mL of distilled water. The brown reaction mixture was vigorously stirred for 30 min at 0 °C (prolonged stirring leads to the formation of not further characterized byproducts), after which the reaction was quenched by the addition of 17.2 mL acetic acid. Then, 6.80 g Na_2SO_3 was carefully added, causing the reaction mixture to turn from brown to olive green immediately. The reaction mixture was diluted with 50% brine solution and the aqueous phase was extracted several times with DCM and ethyl acetate. The combined organic phases were dried over sodium sulfate and after filtering off the desiccant, the solvents were evaporated on the rotary evaporator. A blue-green solid was obtained. The crude product was purified by column chromatography (silica gel, ethyl acetate:*n*-hexane 1:9 to 3:7) to give silicon xanthone **8** (1.75 g, 4.57 mmol, 52% yield) as yellow crystals. – $\mathbf{R_f}$ = 0.29 (ethyl acetate:*n*-hexane 2:8). – **^{1}H NMR** (400 MHz, $CDCl_3$) δ = 8.35 (d, *J*=8.9, 2H), 6.90 (d, *J*=2.8, 2H), 6.85 (dd, *J*=9.0, 2.8, 2H), 3.47 (s, 3H), 3.10 (s, 12H), 0.92 (s, 9H). – **^{13}C NMR** (101 MHz, $CDCl_3$) δ = 185.61, 151.21, 135.49, 131.69, 131.68, 115.08, 113.55, 52.12, 40.20, 25.93, 18.42. – **^{29}Si NMR** (79 MHz, $CDCl_3$) δ = –13.69. – **HRMS** (ESI): *calc.* for $C_{22}H_{31}N_2O_2Si$: 383.2149; *found* 383.2153. – **X-ray** Single crystals of **8** suitable for x-ray diffraction were grown from dissolutions (ethyl acetate:*n*-hexane 2:8) obtained during column chromatography. CCDC 2232514 (**8**) contains the supplementary crystallographic data for this paper. These data can be obtained free of charge from The Cambridge Crystallographic Data Centre via www.ccdc.cam.ac.uk/data_request/cif.

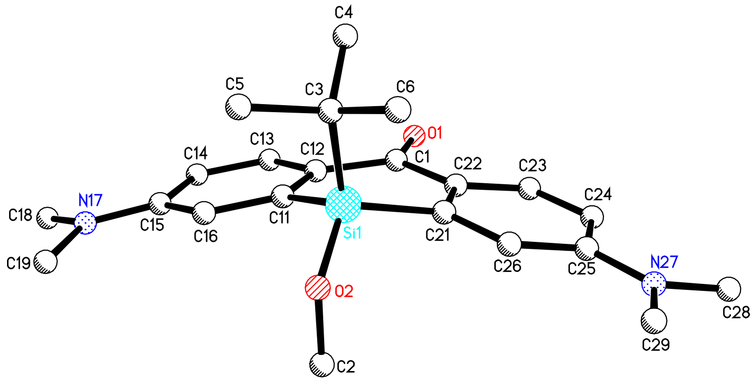

X-ray structure of silicon xanthone **8**.

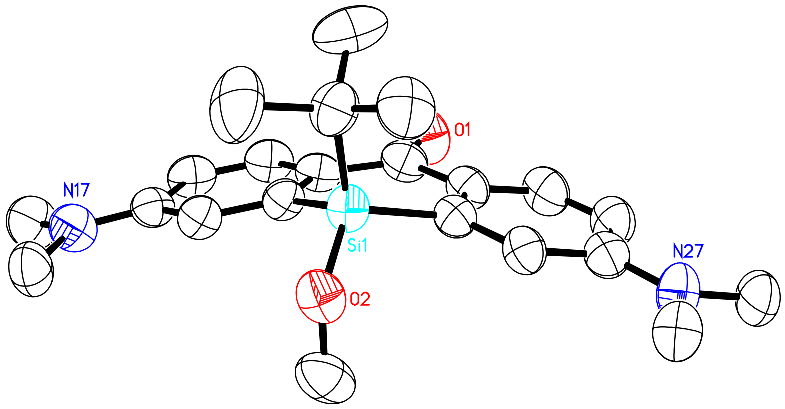

ORTEP presentation of silicon xanthone **8**.

Syntheses of bromo-aryl building blocks 9, 10, 12, and 13

bis((3-methyloxetan-3-yl)methyl)-2-bromoterephthalate

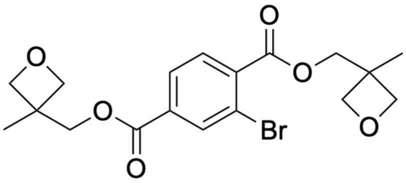

bis((3-methyloxetan-3-yl)methyl)-2-bromoterephthalate

In an oven-dried round bottom flask fitted with a reflux condenser and a septum, bromoterephthalic acid (1.50 g, 6.12 mmol, 1.0 equiv.) was dissolved with 2 drops of DMF under argon and mixed with 10 mL of thionyl chloride. The suspension was refluxed for 3 h, during which the solids went completely into solution. The mixture was evaporated to dryness and concentrated again on the rotary evaporator after the addition of dry DCM. After addition of 10 mL of dry DCM, the solution was added at 0 °C to a mixture of 3-methyl-3-oxetanemethanol (1.31 g, 12.8 mmol, 2.1 equiv.) and pyridine (1.23 mL, 15.2 mmol, 2.5 equiv.) in 20 mL of DCM. The reaction mixture was stirred for 1 h at 0 °C and then overnight at room temperature. The mixture was diluted with DCM and washed with saturated aqueous sodium bicarbonate solution. It was then washed twice with distilled water and then dried with brine solution. Then the organic phase was dried over sodium sulfate and after filtering off, the solvents were removed on rotary evaporator. The crude product was purified via column chromatography (silica gel, ethyl acetate:*n*-hexane 1:1 to pure ethyl acetate, solvents each containing 2% triethylamine) to give bis((3-methyloxetan-3-yl)methyl)-2-bromoterephthalate (2.18 g, 5.28 mmol, 86% yield) as a colorless oil. The acquired analytical data are in accordance with the data in literature (Butkevich, A.N., et al., *Fluorescent rhodamines and fluorogenic carbopyronines for super-resolution STED microscopy in living cells.* Angew Chem Int Ed, 2016. **55**(10): p. 3290-3294).

1,1'-(2-bromo-1,4-phenylen)bis(4-methyl-2,6,7-trioxabicyclo[2.2.2] octane) (9)

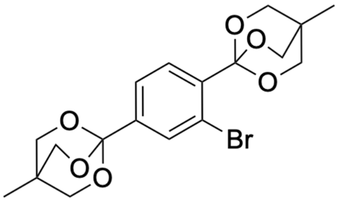

9

In an oven-dried round bottom flask, bis((3-methyloxetan-3-yl)methyl)-2-bromoterephthalate (2.18 g, 5.28 mmol) was dissolved in 10 mL of dry DCM under argon. The solution was cooled to –5 °C and 263 µL of boron trifluoride diethyl etherate was added at this temperature. The mixture was stirred at –5 °C for 1 h and then at room temperature overnight. Subsequently, 732 µL triethylamine was added and the mixture was stirred for 2 h at room temperature. An amorphous precipitate was formed. The mixture was diluted with 17 mL diethyl ether and 8 mL DCM and stirred for an additional 30 min. The suspension was filtered over a Celite pad and the pad was washed with a mixture of diethyl ether and DCM (1:1). The filtrate was concentrated on a rotary evaporator and the crude product was purified via column chromatography (silica gel, ethyl acetate:*n*-hexane 1:3 to ethyl acetate, each solvent containing 2% triethylamine) to give bis-orthoester **9** (469 mg, 1.14 mmol, 22% yield) as a colorless solid. The analytical data obtained are in accordance with the literature (Butkevich, A.N., et al., *Fluorescent rhodamines and fluorogenic carbopyronines for super-resolution STED microscopy in living cells.* Angew Chem Int Ed, 2016. **55**(10): p. 3290-3294).

di-*tert*-butyl-2-bromoterephthalate (10)

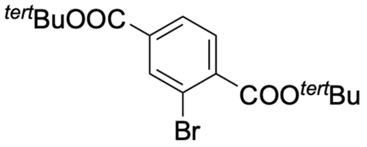

10

In an oven-dried bottom flask fitted with a reflux condenser and a septum, 2-bromoterephthalic acid (1.00 g, 4.08 mmol, 1.0 equiv.) and 4-DMAP (100 mg, 0.815 mmol, 0.2 equiv.) were dissolved under argon in 2.00 mL dry DMF. Di-*tert*-butyl dicarbonate (2.70 g, 12.2 mmol, 3.0 equiv.), dissolved in 10 mL toluene, was added. The suspension was stirred for 20 h at room temperature and then for 40 min at 80 °C oil bath temperature. The reaction was diluted with the addition of saturated sodium bicarbonate solution. The mixture was then extracted several times with a mixture of ethyl acetate and *n*-hexane (2:1). The combined organic phases were washed twice with distilled water and once with brine solution. Then the organic phase was dried over sodium sulfate and after filtering off, the solvents were removed on rotary evaporator. The crude product (1.20 g) was purified via column chromatography (silica gel, *n*-hexane to ethyl acetate:*n*-hexane 2:8) to give di-*tert*-butyl-2-bromoterephthalate (**10**) (1.06 g, 2.80 mmol, 69% yield) as a colorless oil. The analytical data obtained are in accordance with the literature (Butkevich, A.N., et al., *Fluorescent rhodamines and fluorogenic carbopyronines*

for super-resolution STED microscopy in living cells. Angew Chem Int Ed, 2016. **55**(10): p. 3290-3294).

***tert*-butyl-3-bromo-4-methylbenzoate (12)**

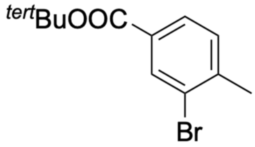

12

In an oven-dried round bottom flask fitted with a reflux condenser and a septum, 3-bromo-4-methylbenzoic acid (500 mg, 2.33 mmol, 1.0 equiv.) and 4-DMAP (29.0 mg, 0.233 mmol, 0.1 equiv.) were dissolved under argon in 1.00 mL dry DMF. Di-*tert*-butyl dicarbonate (764 mg, 3.50 mmol, 1.5 equiv.) dissolved in 5 mL toluene (non-dry) was added. The suspension was refluxed for 5 min and then stirred at 80 °C oil bath temperature for 1 h. The reaction was then stirred overnight at room temperature. The mixture was then diluted with the addition of water and DCM. The aqueous phase was separated and then extracted several times with DCM. Then the combined organic phases were dried over sodium sulfate and after filtering off, the solvents were removed on the rotary evaporator. The crude product (650 mg) was purified via column chromatography (silica gel, diethyl ether:*n*-hexane 2.5:97.5 to 1:9) to give *tert*-butyl-3-bromo-4-methylbenzoate (**12**) (290 mg, 1.07 mmol, 46% yield) as a colorless oil. The acquired characterization data are in accordance with those reported in the literature (Lukinavicius, G., et al., *A near-infrared fluorophore for live-cell super-resolution microscopy of cellular proteins.* Nat Chem, 2013. **5**(2): p. 132-139).

***N*-(3-bromo-4-methylphenyl)-1,1,1-trimethyl-*N*-(trimethylsilyl)silanamine (13)**

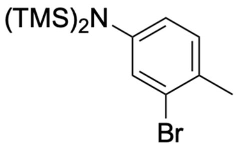

13

Under argon atmosphere, 3-bromo-4-methyl-aniline (390 mg, 2.09 mmol, 1.0 equiv.) was dissolved in dry THF (7.00 mL) in an oven-dried round bottom flask. The brown solution was then cooled to –78 °C and LiHMDS (1.0 M in THF, 4.40 mL, 4.40 mmol, 2.1 equiv.) was added

dropwise. After complete addition of the base, the dark yellow solution was stirred at –78 °C for 10 min and then the cooling bath was removed for 5 min to ensure complete deprotonation. The mixture was cooled down to –78 °C again. Trimethylsilyl chloride (560 µL, 4.39 mmol, 2.1 equiv.) was added dropwise to the yellow solution and then warmed to room temperature. The brown reaction solution was stirred for over four hours. Finally, the solvent was removed under reduced pressure. The remaining brown oily residue was taken up with dry *n*-hexane and filtered. The solvent was removed and the brown oil of crude **13** (778 mg) was used directly for further reaction. The analytical data were in accordance with the published data (P. Shieh, M. S. Siegrist, A. J. Cullen, C. R. Bertozzi, *Proceedings of the National Academy of Sciences of the United States of America* **2014**, *111*, 5456).

Syntheses of the silicon rhodamines 15, 16, 17, 18, 19, 20, and 21

N-(5-(tert-Butyl)-7-(dimethylamino)-5-methoxy-10-phenyldibenzo[b,e]silin-3(5H)-yliden)-N-methylmethanaminium chloride (15)

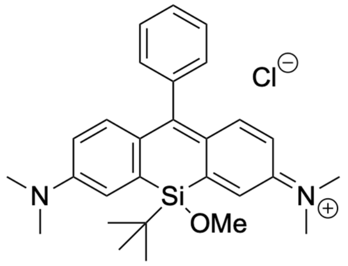

15

In a heated round bottom flask, silicon xanthone **8** (100 mg, 0.261 mmol, 1.0 equiv.) was dissolved in 10 mL of dry THF under argon. The solution was cooled to –78 °C, then phenyllithium (1.9 M in dibutyl ether, 1.39 mmol, 731 µL, 5.0 equiv.) was added at this temperature. The reaction mixture turned a deep red color. The cold bath was removed, allowing the reaction mixture to warm up to room temperature. The mixture was stirred for 2 h under light protection. Distilled water was then added and a few milliliters of 1 N hydrochloric acid was added until the organic phase turned deep blue. The aqueous phase was extracted several times with DCM. The combined organic phases were dried over sodium sulfate and after filtering off, the solvents were removed on the rotary evaporator. The crude material (188 mg) was purified via column chromatography (silica gel, methanol:dichloromethane 0.5:99.5 to 1:9) to give dye **15** (101.3 mg, 0.211 mmol, 81% yield) as a blue powder. – **R_f** = 0.63 (acetonitrile:water 2:1, reversed-phase C_{18} thin-layer chromatography). – **^{1}H NMR** (400 MHz, CD_3OD) δ = 7.59 – 7.53 (m, 3H), 7.32 (d, *J*=2.9, 2H), 7.30 – 7.18 (m, 2H), 7.16 (d, *J*=9.7, 2H), 6.81 (dd, *J*=9.7, 2.9, 2H), 3.61 (s, 3H), 3.35 (s, 12H), 0.97 (s, 9H). – **^{13}C NMR** (101 MHz, CD_3OD) δ = 170.98, 155.16, 144.19, 143.61, 140.12, 130.70, 129.98, 129.48, 129.26, 122.74, 115.25, 52.87, 40.99, 25.12, 19.23. – **^{29}Si NMR** (79 MHz, CD_3OD) δ = 13.51. – **HRMS** (ESI): *calc.* for $C_{28}H_{35}N_2OSi^+$: 443.2513; *found* 443.2517.

(*note: the silanol methyl ether **15** was purified via HPLC to acquire the NMR data, however, the methyl ether decomposed/was cleaved slowly under acidic conditions after using TFA (0.1 %) as additive in acetonitrile and in deionized water as HPLC solvents*).

N-(5-(tert-butyl)-7-(dimethylamino)-5-hydroxy-10-phenyldibenzo[b,e] silin-3(5H)-ylidene)-N-methylmethanaminium trifluoroacetate (16)

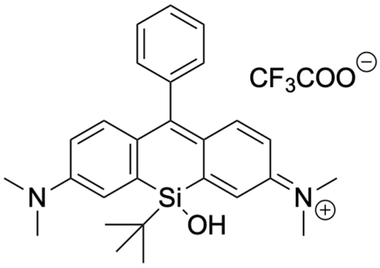

16

In an oven-dried round bottom flask, silicon xanthone **8** (150 mg, 0.392 mmol, 1.0 equiv.) was dissolved in 15 mL of dry THF under argon. The solution was cooled to –78 °C, then phenyllithium (1.9 M in dibutyl ether, 2.08 mmol, 1.01 mL, 5.0 equiv.) was added at this temperature. The reaction mixture turned to a deep red color. The cold bath was removed, allowing the reaction mixture to warm up to room temperature. The mixture was stirred overnight under light protection. Distilled water was then added and a few milliliters of 1 N hydrochloric acid was added dropwise until the organic phase turned deep blue. The aqueous phase was extracted several times with DCM. The combined organic phases were dried over sodium sulfate and, after filtration, the solvents were removed on the rotary evaporator. The crude product **15** (333 mg) was further used without purification.
After the crude product **15** (333 mg) was dissolved in 20 mL of DCM, 4.1 mL of TFA was added at 0 °C. The reaction mixture was stirred for two days protected from light and allowed to warm up to room temperature. Subsequently, the solvents were removed at the rotary evaporator and the crude product was evaporated two more times with toluene on the rotary evaporator to remove residues of TFA. The crude product (353 mg) was purified via column chromatography (silica gel, methanol:dichloromethane 2.5:97.5 to 60:40). The blue fractions were collected and the solvent was evaporated to give 296 mg of a blue powder. The crude material showed two peaks in HPLC, silanol **15** and methyl ether **16** in a ratio of 3:1. 20 mg of the crude product were purified again by reversed-phase column chromatography (C_{18} silica gel, acetonitrile (containing 0.1% TFA):water (with 0.1% TFA) 1:9 to 6:4) to give silicon rhodamine **16** (9.9 mg, 0.0182 mmol, equivalent to 69% yield at full purification) as a blue powder. – **R*f*** = 0.58 (acetonitrile:water 2:1, reversed-phase C_{18} TLC). – **^{1}H NMR** (400 MHz, $CDCl_3$) δ = 7.57 – 7.45 (m, *J*=2.8, 5H), 7.25 – 7.09 (m, 2H), 7.07 (d, *J*=9.6, 2H), 6.50 (dd, *J*=9.6, 2.8, 2H), 3.32 (s, 12H), 0.95 (s, 9H). – **HRMS** (ESI): *calc.* for $C_{27}H_{33}N_2OSi^+$: 429.2357; *found.* 429.2356.

_N_-(10-(5-(_tert_-butoxycarbonyl)-2-methylphenyl)-5-(_tert_-butyl)-7-(di-methylamino)-5-methoxydibenzo[_b_,_e_]silin-3(5_H_)-ylidene)-_N_-methyl-methanaminium chloride (17)

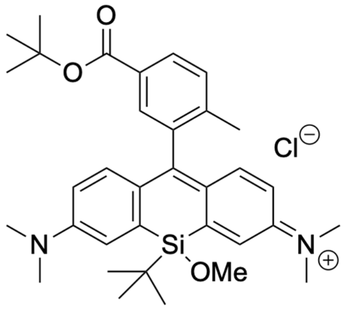

17

Under argon, 3-bromo-4-methyl-benzoic acid tert-butyl ester (**12**) (410 mg, 1.52 mmol, 5.8 equiv.) was dissolved in 10 mL of dry THF in an oven-dried round bottom flask. The solution was cooled to –78 °C, then *tert*-BuLi (1.7 M in pentane, 3.06 mmol, 1.80 mL, 11.6 equiv.) was added dropwise at this temperature. The yellow reaction mixture was stirred at –78 °C for 30 min. Silicon xanthone **8** (100 mg, 0.261 mmol, 1.0 equiv.), dissolved in 5.00 mL dry THF, was added at this temperature so that the reaction mixture turned deep red. The cooling bath was removed, allowing the reaction mixture to warm up to room temperature. The mixture was stirred under light exclusion overnight. Distilled water and DCM were then added and a few milliliters of 1 N hydrochloric acid was added until the organic phase turned deep blue. The organic phase was separated and the aqueous phase was extracted a few times with DCM. The combined organic phases were dried with brine solution and over sodium sulfate, and after filtering off, the solvents were removed on the rotary evaporator. The crude product (474 mg) was purified via column chromatography (silica gel, methanol:dichloromethane 0.5:99.5 to 1:9) to give product **17** (72.7 mg, 0.123 mmol, 47% yield) as a blue solid. NMR revealed the rotamers **17a** (major rotamer) and **17b** (minor rotamer). NOE measurements verified the structure of **17a** and **17b**. – $\mathbf{R}_{\boldsymbol{f}}$ = 0.26 (acetonitrile:water 2:1, reversed-phase C_{18} TLC). – **^{1}H NMR** (400 MHz, CD_3OD) δ = 8.06 (dd, *J* = 8.0, 1.8 Hz, 1H, a+b), 7.61 (dd, *J* = 8.5, 1.8 Hz, 1H, a+b), 7.53 (dd, *J* = 8.1, 2.6 Hz, 1H, a+b), 7.36 (dd, *J* = 4.3, 2.8 Hz, 2H, a+b), 7.03 (t, *J* = 9.3 Hz, 2H, a+b), 6.84 (dd, *J* = 9.7, 2.9 Hz, 2H, a+b), 3.64 (s, 3H, b), 3.63 (s, 3H, a), 3.38 (s, 12H, a+b), 2.16 (s, 3H, a), 2.09 (s, 3H, b), 1.58 (s, 9H, a), 1.57 (s, 9H, b), 1.01 (s, 9H, a+b). – **^{13}C NMR** (101 MHz, CD_3OD) δ = 168.96, 168.81, 166.53, 166.49, 155.35, 155.33, 144.16, 144.10, 142.78, 142.42, 142.36, 141.96, 140.11, 139.77, 131.84, 131.68, 131.60, 131.06, 130.90, 130.04, 129.92, 129.87, 123.16, 122.99, 115.86, 115.84, 82.78, 54.81, 52.90, 41.07, 28.38, 28.33, 25.27, 25.22, 25.10, 19.93, 19.59, 19.48, 19.30. – **HRMS** (ESI): *calc.* for $C_{34}H_{45}N_2O_3Si^{+}$: 557.3194; *found* 557.3200.

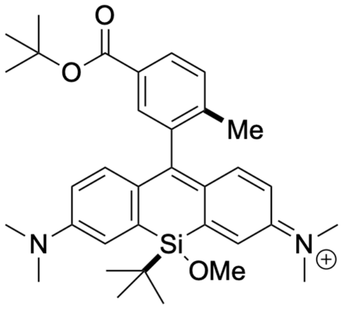

17a

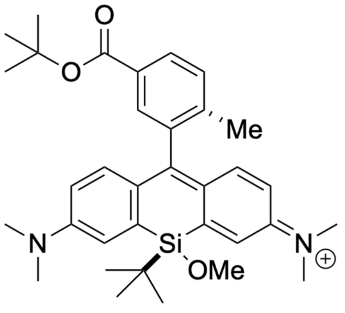

17b

3-(5-(*tert*-butyl)-7-(dimethylamino)-3-(dimethylimine)-5-hydroxy-3,5-dihydrodibenzo[*b,e*]silin-10-yl)-4-methylbenzoate (18)

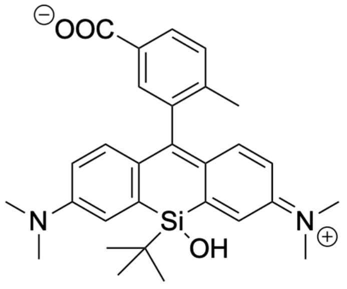

18

Under argon, 3-bromo-4-methyl-benzoic acid tert-butyl ester **12** (410 mg, 1.52 mmol, 5.8 equiv.) was dissolved in 10 mL of dry THF in an oven-dried round bottom flask. The solution was cooled to –78 °C, then *tert*-BuLi (1.7 M in pentane, 3.06 mmol, 1.80 mL, 11.6 equiv.) was added at this temperature. The yellow reaction mixture was stirred at –78 °C for 30 min. Silicon xanthone **8** (100 mg, 0.261 mmol, 1.0 equiv.), dissolved in 5.00 mL dry THF, was added at this temperature and the reaction mixture turned deep red. The cold bath was removed, allowing the reaction mixture to warm up to room temperature. The mixture was stirred under light protection overnight. Distilled water and DCM were then added and a few milliliters of 1 N hydrochloric acid was added dropwise until the organic phase turned deep blue. The organic phase was separated and the aqueous phase was extracted a few times with DCM. The combined organic phases were dried over sodium sulfate and after filtering off, the solvents were removed on the rotary evaporator. The crude material **17** (470 mg) was further used without purification.

After crude material **17** (470 mg) was dissolved in 20 mL DCM, 4.1 mL TFA was added at 0 °C. The color of the reaction mixture changed from blue to bright yellow-green. The reaction mixture was stirred overnight, protected from light, and allowed to warm up to room temperature. The next day, the solvents were removed at the rotary evaporator and the crude

product was evaporated two more times with toluene at the rotary evaporator. The crude product (510 mg) was purified via column chromatography (silica gel, methanol:dichloromethane 2.5:97.5 to 60:40). The blue fractions were collected and the solvent evaporated off to give 441 mg of a blue powder. The material was purified again by reversed-phase column chromatography (C_{18} silica gel, acetonitrile (containing 0.1% TFA):water (containing 0.1% TFA) 6:4) to give silicon rhodamine **18** (56.1 mg, 0.115 mmol, 44% yield) as a blue powder. The silicon rhodamine could be separated by prep. HPLC into two diastereomers (ratio 2:1, most likely rotamers as they were equilibrating) showing the same mass in MS (performed on an Interchim puriFlash 4250 2X preparative HPLC/Flash hybrid system (article No. 1I5140, Interchim) with a 2 mL injection loop, a 200–600 nm UV-Vis detector and an integrated ELSD detector (article No. 1A3640, Interchim); preparative column: Interchim Uptisphere Strategy C18-HQ, 10 µm, 250×21.2 mm (article No. US10C18HQ-250/212, Interchim), typical flow-rate: 20 mL/min; gradient: acetonitrile (containing 0.1% TFA):water (containing 0.1% TFA) 40:60 to 100:0). The spectroscopic data collected describe the major component (extracted H-NMR data from a mixture of rotamers). – $\mathbf{R_f}$ = 0.23 (acetonitrile:water 2:1, reversed-phase C_{18} TLC). – $\mathbf{^1H}$ **NMR** (400 MHz, $CDCl_3$+drops CD_3OD) δ = 7.95 (dd, *J*=8.0, 1.9, 1H), 7.53 (d, *J*=1.8, 1H), 7.33 – 7.28 (m, 1H), 7.24 – 7.20 (m, 2H), 6.87 (d, *J*=9.6, 2H), 6.48 (dd, *J*=9.6, 2.8, 2H), 3.20 (s, 12H), 2.00 (s, 3H), 0.83 (s, 9H). – $\mathbf{^{29}Si}$ **NMR** (79 MHz, $CDCl_3$+drops CD_3OD) δ = –18.73. – **HRMS** (ESI): *calc.* for $C_{29}H_{35}N_2O_3Si$: 487.2411; *found* 487.2414.

_N_-(10-(5-amino-2-methylphenyl)-5-(_tert_-butyl)-7-(dimethylamino)-5-methoxydibenzo [_b_,_e_]silin-3(5_H_)-ylidene)-_N_-methylmethanaminium chloride (**19**)

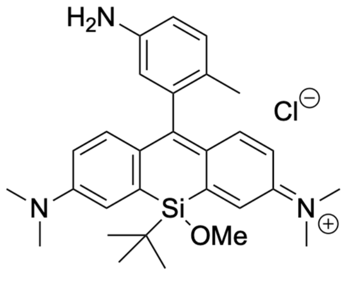

19

The silicon rhodamine **19** was synthesized analogue to rhodamine **17**. The bromo aryl building block **13** (778 mg, 2.35 µmol, 7.0 equiv.) was lithiated with *tert*-butyllithium (1.7 M in pentane, 2.80 mL, 4.76 mmol, 14.2 equiv.). Into this solution was injected a solution of silicon xanthone **8** (129 mg, 336 µmol, 1.0 equiv.) in 10 mL of anhydrous THF. After column chromatography purification (silica gel, methanol:dichloromethane 95:5 to 80:20), silicon rhodamine **19**

(100 mg, 197 μmol, 59% yield) was obtained as a blue solid. – **R$_f$** = 0.57 (methanol:dichloromethane 10:90) – **^{1}H NMR** (400 MHz, d_4-MeOD) δ = 7.34–7.22 (m, 4H), 7.10 (d, J = 8.2 Hz, 1H), 6.85 (d, J = 2.9 Hz, 1H), 6.84–6.79 (m, 2H), 6.47 (t, J = 2.3 Hz, 1H), 3.61 (s, 3H), 3.36 (s, 12H), 1.91 (s, 3H), 0.99 (s, 9H). – **^{13}C NMR** (101 MHz, d_4-MeOD) δ = 172.2, 155.3, 146.7, 144.1, 143.3, 140.0, 132.0, 130.3, 125.2, 122.7, 117.1, 116.0, 115.5, 52.5, 41.0, 25.3, 19.4, 18.7. – **HRMS** (ESI): *calc.* for $C_{29}H_{38}N_3OSi^+$: 472.2779; *found* 472.2779.

***N*-(5-(*tert*-butyl)-7-(dimethylamino)-5-methoxy-10-(2-(methylthio)phenyl) dibenzo[*b*,*e*]silin-3(5*H*)-ylidene)-*N*-methylmethanaminium chloride (20)**

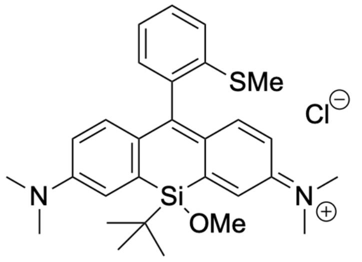

20

The silicon rhodamine **20** was synthesized analogue to rhodamine **17**. In an oven-dried round-bottom flask, 2-bromothioanisole **14** (372 mg, 1.83 μmol, 7.0 equiv.) was lithiated with *tert*-butyllithium (1.7 M in pentane, 2.20 mL, 3.74 mmol, 14.3 equiv.). Into this solution was injected a solution of silicon xanthone **8** (100 mg, 261 μmol, 1.0 equiv.) in anhydrous THF . After purification by column chromatography (silica gel, methanol:dichloromethane 95:5 to 85:15), 49 mg (94 μmol, 36% yield) of silicon rhodamine **20** was obtained as a blue solid. – **R$_f$** = 0.62 (methanol:dichloromethane 10:90) – **^{1}H NMR** (400 MHz, d_4-MeOD) δ = 7.62–7.48 (m, 2H), 7.42–7.30 (m, 3H), 7.15– 7.03 (m, 3H), 6.85–6.74 (m, 2H), 3.60 (s, 3H), 3.36 (s, 12H), 2.37 (s, 3H), 1.01 (s, 9H). – **^{13}C NMR** (101 MHz, d_4-MeOD) δ = 169.3, 155.2, 144.0, 142.9, 130.8, 130.5, 130.2, 129.7, 127.0, 126.0, 122.7, 115.5, 41.0, 25.5, 25.2, 19.2, 16.0. – **HRMS** (ESI): *calc.* for $C_{29}H_{37}N_2OSSi^+$: 489.2390; *found* 489.2382.

***N*-(10-(5-azido-2-methylphenyl)-5-(*tert*-butyl)-7-(dimethylamino)-5-hydroxydibenzo[*b*,*e*]silin-3(5*H*)-ylidene)-*N*-methylmethanaminium chloride (21)**

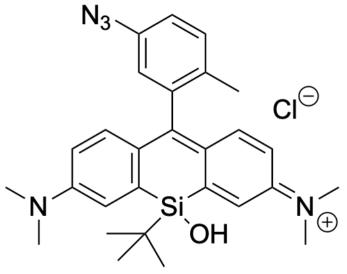

21

In a flask, the amino silicon rhodamine **19** (150 mg, 295 µmol, 1.0 equiv.) was dissolved in acetic acid (3.0 mL) and distilled water (1.0 mL) and cooled down to 0 °C. Sodium nitrite (30.5 mg, 443 µmol, 1.5 equiv.) in 500 µL water was slowly added and the blue solution turned greenish. After 15 minutes at 0 °C, sodium azide (38.4 mg, 590 µmol, 2.0 equiv.) dissolved in 500 µL water was slowly added. Subsequently, the resulting deep blue solution was stirred at 0 °C for 1 h and then at room temperature for 1.5 hours. Then, the solvent was completely removed in vacuo. The crude product was purified by column chromatography (silica gel, dichloromethane:methanol 95:5 to 80:20) to afford the azido silicon rhodamine **21** (139 mg, 268 µmol, 91%) as a deep blue dye. – **R_f** = 0.48 (methanol:dichloromethane 10:90) – **^{1}H NMR** (400 MHz, d_4-MeOD) δ = 7.47–7.39 (m, 3H), 7.20 (t, *J* = 9.1 Hz, 1H), 7.07 (t, *J* = 10.7 Hz, 2H), 6.81 (d, *J* = 9.0 Hz, 2H), 6.71 (d, *J* = 21.7 Hz, 1H), 3.37 (s, 12H), 1.96 (s, 3H), 0.99 (s, 9H). – **^{13}C NMR** (101 MHz, d_4-MeOD) δ = 175.9, 155.3, 147.2, 142.3, 142.2, 133.1, 130.0, 129.5, 122.8, 121.6, 120.5, 119.7, 115.4, 41.0, 25.3, 21.3, 19.2. – **HRMS** (ESI): *calc.* for $C_{28}H_{34}N_5OSi^+$: 484.2527; *found* 484.2526.

Syntheses of silyl fluorides 24, 28, and of silanol 25

5-(*tert*-butyl)-3,7-bis(dimethylamin)-5-fluorodibenzo[*b*,*e*]silin-10(5*H*)-one (24)

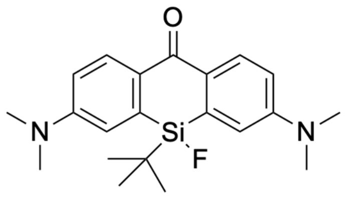

24

In a Teflon vessel (Falcon™ centrifuge tube), silicon xanthone **8** (85.0 mg, 0.222 mmol) was dissolved in 6.00 mL dry THF under argon, and Olah reagent (70% HF, 30% pyridine, 600 µL) was added dropwise. The color of the solution changed from yellow to orange. The reaction mixture was stirred at room temperature for one day and was diluted then with distilled water and DCM was added. The phases were separated and the aqueous phase was extracted several times with DCM. The combined organic phases were dried with sodium sulfate. After filtering off the desiccant, the solvents were evaporated off on a rotary evaporator to give silicon fluoride **24** (82.0 mg, 0.221 mmol, quant. yield) as a yellow oil that showed no impurities in HPLC. – $\mathbf{R}_f$ = 0.31 (ethyl acetate:*n*-hexane 2:8). – ^{1}**H NMR** (400 MHz, $CDCl_3$) δ = 8.32 (dd, *J*=9.0, 1.0, 2H), 6.94 (d, *J*=2.8, 2H), 6.87 (dd, *J*=9.0, 2.8, 2H), 3.10 (s, 12H), 1.00 (s, 9H). – ^{13}**C NMR** (101 MHz, $CDCl_3$) δ = 185.07, 151.29, 133.95, 131.65, 130.73, 114.90, 114.03, 40.16, 25.49, 19.09. – ^{29}**Si NMR** (79 MHz, $CDCl_3$) δ = –6.86 (d, *J*=304.3). – ^{19}**F NMR** (376 MHz, $CDCl_3$) δ = –187.05. – **HRMS** (ESI): *calc.* for $C_{21}H_{28}FN_2OSi^+$: 371.1949; *found* 371.1955.

5-(*tert*-butyl)-3,7-bis(dimethylamino)-5-hydroxydibenzo[*b*,*e*]silin-10(5*H*)-one (25)

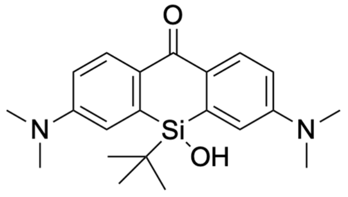

25

In a Teflon vessel (Falcon™ centrifuge tube), silicon xanthone **8** (56.3 mg, 0.147 mmol, 1.0 equiv.) was dissolved in 3.50 mL dry THF under argon, then TBAF (1.0 M in THF, 0.163 mmol, 163 µL, 1.1 equiv.) was added dropwise. The yellow solution was stirred at room temperature for one day and was diluted then with distilled water and DCM. The phases were separated and the aqueous phase was extracted several times with DCM and ethyl acetate.

The combined organic phases were washed with brine and dried with sodium sulfate. After filtering off the desiccant, the solvents were evaporated on the rotary evaporator. Purification by column chromatography (silica gel, ethyl acetate:*n*-hexane 3:7 to 1:1) afforded silanol **25** (41.8 mg, 0.113 mmol, 77% yield) as yellow crystals. **R_f** = 0.51 (ethyl acetate:*n*-hexane). – **^{1}H NMR** (400 MHz, $CDCl_3$) δ = 8.31 (d, *J*=9.0, 2H), 6.96 (d, *J*=2.8, 2H), 6.84 (s, 2H), 3.09 (s, 12H), 2.17 (s, 1H), 0.95 (s, 9H). – **^{13}C NMR** (101 MHz, $CDCl_3$) δ = 185.61, 151.29, 137.68, 131.42, 130.88, 115.29, 113.42, 40.19, 25.89, 18.69. – **^{29}Si NMR** (79 MHz, $CDCl_3$) δ = –16.47. – **HRMS** (ESI neg): *calc.* for $C_{21}H_{27}N_2O_2Si^-$: 367.1842; *found* 367.1887; (ESI pos): *calc.* for $C_{21}H_{29}N_2O_2Si^+$: 369.1998; *found* 369.1999.

_N_-(5-(_tert_-butyl)-7-(dimethylamino)-5-fluoro-10-(((trifluoromethyl) sulfonyl)oxy)dibenzo[_b,e_]silin-3(5_H_)-yliden)-_N_-methylmethanaminium trifluoromethanesulfonate (28)

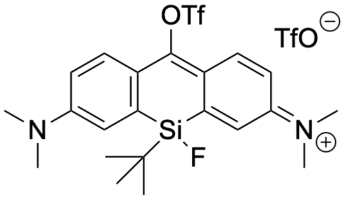

28

In an oven-dried round bottom flask, the fluorinated silicon xanthone **24** (11.0 mg, 0.0297 mmol, 1.0 equiv.) was dissolved in 1.00 mL dry acetonitrile under argon. Triflic anhydride solution (1 M in DCM, 0.0327 mmol, 33 µL, 1.1 equiv.) was then added at room temperature. The deep blue reaction mixture was stirred for 20 h at room temperature. Then the solvent was evaporated by using a rotary evaporator. The residue (quant. yield) was dissolved in $CDCl_3$, filtered, and analyzed by NMR spectroscopy. – **^{1}H NMR** (400 MHz, $CDCl_3$) δ = 8.44 (dd, *J*=9.8, 0.8, 1H), 7.90 (d, *J*=9.7 Hz, 1H), 7.27 (d, *J*=2.7, 1H), 7.19 (d, *J*=2.8, 1H), 7.01 (dd, *J*=9.7, 2.7, 1H), 6.99 (dd, *J*=10.1, 2.9, 1H), 3.49 (s, 6H), 3.45 (s, 6H), 1.03 (s, 9H). – **^{19}F NMR** (376 MHz, $CDCl_3$) δ = –78.57, –81.16, –187.24.

Synthesis of silicon pyronines 30, 31, and [^{19}F]31

10-(benzylimino)-5-(*tert*-butyl)-5-methoxy-*N*^{3},*N*^{3},*N*^{7},*N*^{7}-tetramethyl-5,10-dihydrodibenzo[*b*,*e*]silin-3,7-diamine (30)

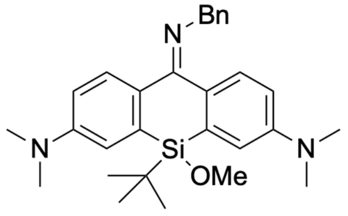

30

Silicon xanthone **8** (10.0 mg, 0.0262 mmol, 1.0 equiv.) was dissolved in 500 µL of dry DCM under argon in a round bottom flask. Triflic anhydride solution (1 M in DCM, 39.3 µmol, 39 µL, 1.5 equiv.) was then added at room temperature. The deep blue reaction mixture was stirred for 20 min at room temperature. Then the mixture was added dropwise at 0 °C to a solution of benzylamine (8.4 mg, 0.0784 mmol, 3.0 equiv.) and 2,6-lutidine (17.0 mg, 159 µmol, 6.0 equiv.) in 500 µL dry DCM. The color of the mixture changed from deep blue to bright orange upon addition. The mixture was stirred at 0 °C for 30 min, then saturated sodium hydrogen carbonate solution was added. After the phases were separated, the aqueous phase was extracted several times with DCM. The combined organic extracts were dried over sodium sulfate and after filtering off the solvent, the filtrate was concentrated on the rotary evaporator. Purification via preparative thin-layer chromatography (silica gel, methanol:DCM 5:95, the solvent front was allowed to reach the top of the silica gel plate three times, detachment from the silica gel with 10% methanol in DCM) afforded pyronine **30** (9.5 mg, 0.0201 mmol, 77% yield) as an orange solid. – **R*f*** = 0.48 (methanol:dichloromethane 10:90). – **^{1}H NM**R (400 MHz, $CDCl_3$) δ = 8.42 (d, *J*=8.5, 1H), 7.51 – 7.44 (m, 2H), 7.35 (t, *J*=7.5, 2H), 7.26 (d, *J*=3.9, 2H), 7.00 (d, *J*=2.8, 1H), 6.94 (dd, *J*=8.9, 2.8 Hz, 1H), 6.88 (d, *J*=2.7, 1H), 6.68 (dd, *J*=8.9, 2.8, 1H), 5.27 (d, *J*=14.7, 1H), 4.98 (d, *J*=14.7, 1H), 3.42 (s, 3H), 3.10 (s, 6H), 3.07 (s, 6H), 0.90 (s, 9H). – **^{13}C NMR** (101 MHz, $CDCl_3$) δ = 151.17, 150.82, 136.29, 134.85, 131.23, 130.79, 128.90, 127.77, 127.58, 116.76, 115.71, 114.08, 111.55, 70.61, 52.14, 40.20, 40.10, 29.75, 25.30, 18.51. – **^{29}Si NMR** (79 MHz, $CDCl_3$) δ = –11.57. – HRMS (ESI): *calc.* for $C_{29}H_{38}N_3OSi^+$: 472.2779; *found* 472.2779. – **X-ray** Single crystals of **30** suitable for x-ray diffraction were grown from a saturated solution in dichloromethane and methanol upon standing and low evaporation at room temperature. CCDC 2232515 (**30**) contains the supplementary crystallographic data for this paper. These data can be obtained free of charge from The Cambridge Crystallographic Data Centre via www.ccdc.cam.ac.uk/data_request/cif.

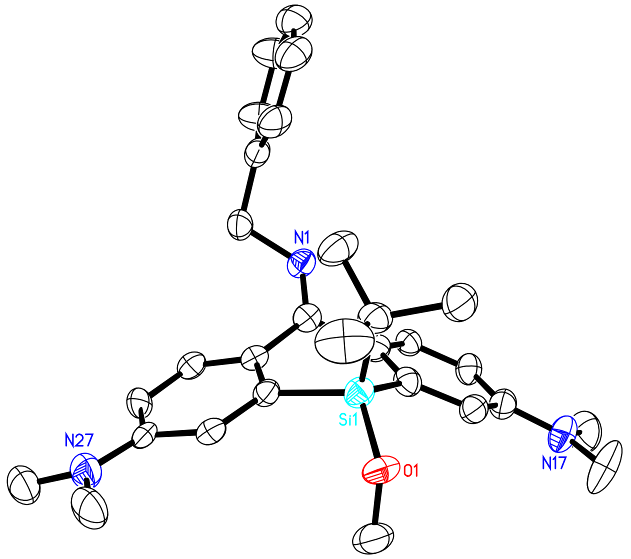

ORTEP presentation of the structrue of pyronine **30**.

Fluorination of silicon pyronine 30 to 31

10-(benzylimino)-5-(*tert*-butyl)-5-fluoro-N^3,N^3,N^7,N^7-tetramethyl-5,10-dihydrodibenzo[*b*,*e*]siline-3,7-diamine (31)

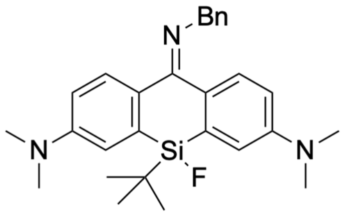

31

Silicon pyronine **30** (8.0 mg) was dissolved in 1.00 mL anhydrous THF under argon. Then, 50 µL of Olah reagent (70% HF, 30% pyridine) was added at 0 °C whereby the color changed from yellow to orange. The mixture was stirred for 20 min, then the ice bath was removed and the mixture was stirred for 20 min at rt. Then the reaction was quenched by the addition of water. The water phase was extracted three times with DCM. The organic phase was dried with sodium sulfate, filtrated and the solvents were evaporated *in vacuo* to afford crude material of fluoro-pyronine **31** that showed sufficient purity in HPLC. – $\mathbf{R_f}$ = none (decomposing on silica gel and on alumina) – ^{1}**H NMR** (400 MHz, $CDCl_3$) δ = 8.64 (d, J = 9.0 Hz, 1H), 7.63 (d, J = 9.0 Hz, 1H), 7.55 – 7.46 (m, 2H), 7.37 – 7.28 (m, 3H), 7.07 – 6.96 (m, 2H), 6.90 (d, J = 2.7 Hz, 1H), 6.71 (dd, J = 9.1, 2.8 Hz, 1H), 5.39 (dd, J = 14.4, 6.5 Hz, 1H), 5.11 (dd, J = 14.4, 5.9 Hz, 1H), 3.14 (s, 6H), 3.09 (s, 6H), 0.94 (d, J = 1.2 Hz, 7H). – ^{13}**C NMR** (101 MHz, $CDCl_3$) δ = 172.64, 151.81, 151.50, 135.88, 131.95, 131.68, 129.05, 128.31, 128.18, 125.57, 124.98, 120.81, 120.78, 116.56, 115.53, 114.61, 112.09, 68.03, 40.05, 30.37, 29.75, 25.66, 24.70, 18.86, 18.70. – ^{19}**F NMR** (376 MHz, $CDCl_3$) δ –187.20 – **HRMS** (ESI): *calc.* for $C_{28}H_{35}FN_3Si$: 460.2579; *found* 460.2580.

Radiolabelling of 31 via SiFA-IE

Production of fluorine-18: The nuclide was produced via an $^{18}O(p, n)^{18}F$ reaction by bombardment of enriched [^{18}O]water with 16.5 MeV protons using a PETtrace 890™ Cyclotron (GE Healthcare, Chicago, USA) from the University Hospital at the Eberhard Karls University in Tübingen, Germany.

Preparation of aqueous Kryptofix® 2.2.2/K_2CO_3/acetonitrile solution: In a vial Kryptofix® 2.2.2 (5.00 mg, 13.3 µmol) was dissolved in 50 µL of an aqueous potassium carbonate solution (20 mg/mL). After complete dissolution of Kryptofix® 2.2.2, acetonitrile (1 mL) was added to the flask (Vial 1).

Preparation of anhydrous fluoride-18: The target water containing [^{18}F]F^- was trapped on a QMA-cartridge by passing the irradiated and fluoride containing solution of [^{18}O]H_2O through a QMA-cartridge. Subsequently, the trapped [^{18}F]F^- was eluted with the aqueous solution of potassium carbonate and acetonitrile (Vial 1) into the reaction vessel. At 100 °C and by using vacuum and a nitrogen flow, the vial containing [^{18}F]F^- was dried.

The precursor **31** (1.0 mg, 2.12 µmol) was dissolved in anhydrous acetonitrile (212 µL) to achieve a final precursor concentration of 10.0 mM. Then, **31** (solution in acetonitrile) (100 µL, 1.00 µmol) was added to a sealable conical flask containing anhydrous [^{18}F]KF (100 MBq). Afterwards, the conical flask was sealed and warmed up to 40 °C for 10 minutes. Subsequently an aliquot was taken, diluted, and then injected into the radio-HPLC to determine the radiochemical conversions. The partition coefficient was determined by using the 2-phase system in *n*-octanol and PBS (pH=7.4; n=10) according to the literature [2]

For human serum stability studies, the vial containing [^{18}F]**31** in acetonitrile was evaporated under a gentle flow of nitrogen. Then the residue was redissolved in DMSO and PBS (pH=7.4). This solution was then added to human serum and the in vitro stability was analyzed via radio-HPLC after several time points.

[2] A. A. Wilson, L. Jin, A. Garcia, J. N. DaSilva, S. Houle, *Appl. Radiat. Isot.* **2001**, *54*, 203–208.

Original HPLC data (see general remarks for details on HPLC parameters and setup)

[^{19}F]**31** (UV), non-radioactive standard

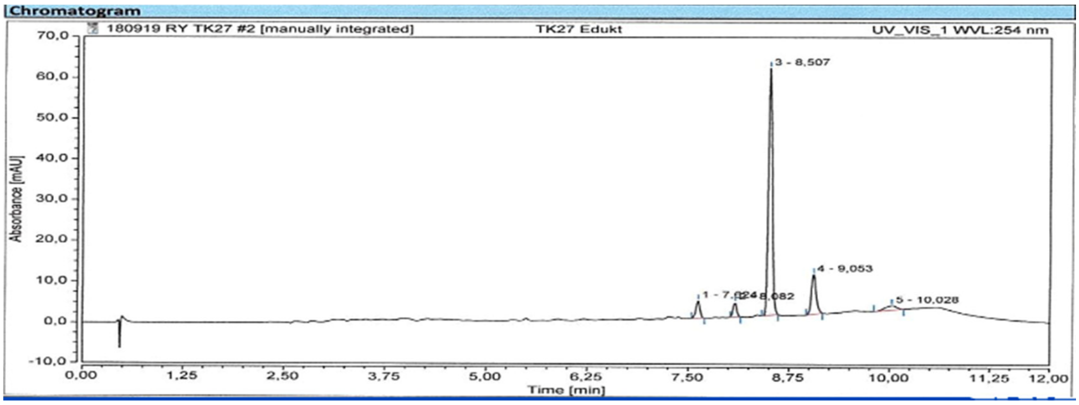

[^{18}F]**31** (radio-HPLC), radioactive compound (non-optimized conditions), radiochemical conversion (RCC) of approx. 30%, free fluoride at 0.73 min.

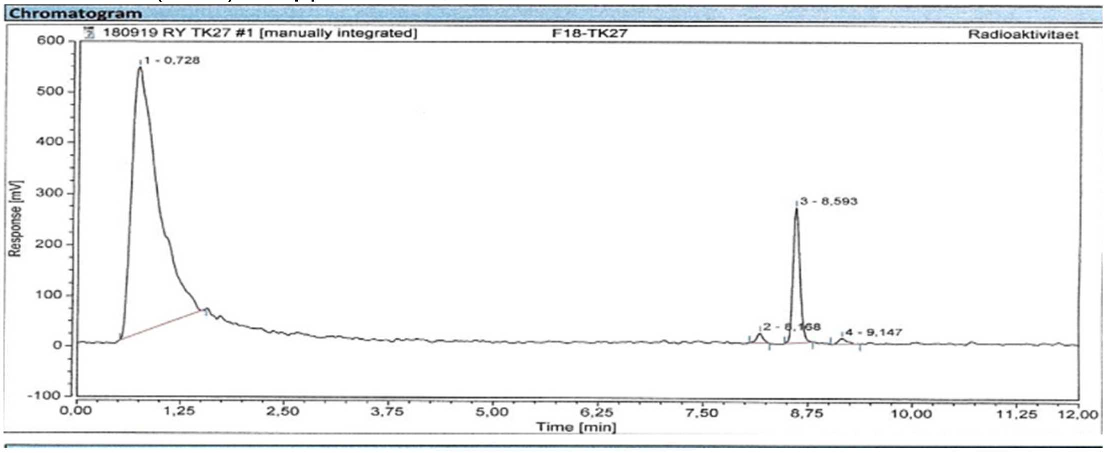

[^{18}F]**31** (radio-HPLC), radioactive compound (optimized conditions), RCC of approx. 70%, free fluoride at 0.73 min.

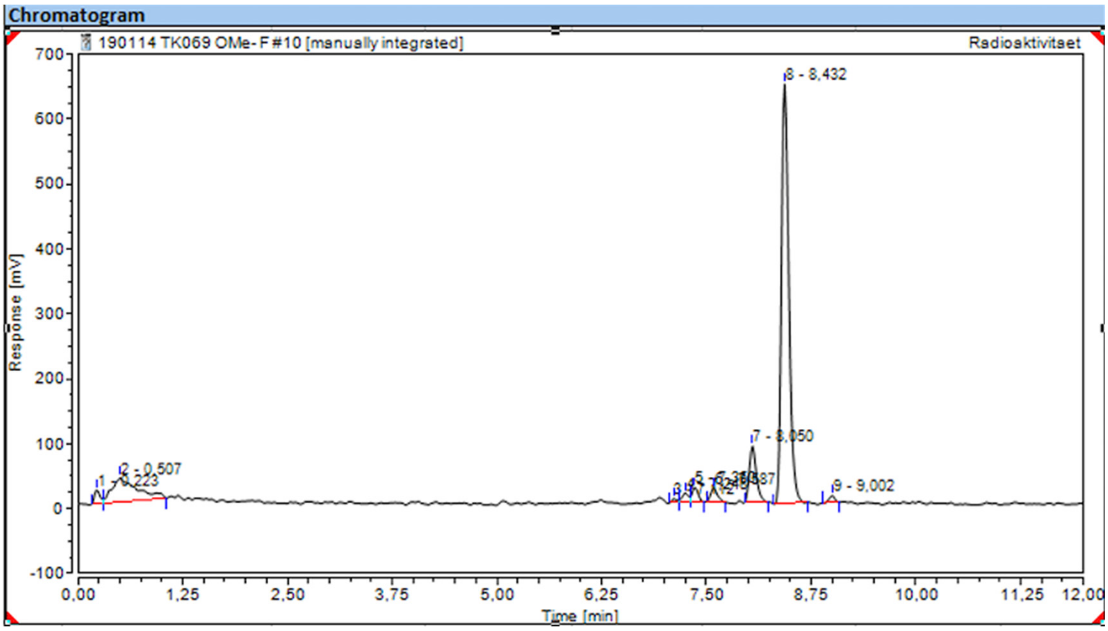

Optical spectroscopy

4.1 Absorption and emission

The dyes were dissolved in H_2O, PBS, MeCN or MeOH, diluted in the respective solvent to the low µM range for UV/Vis/NIR absorbance measurements and to the high nM range for fluorescence emission measurements, and measured in standard 1 cm quartz cuvettes. Absorbance was measured with a Varian Cary 500 UV-VIS NIR Spectrophotometer (Agilent). Fluorescence emission was measured with a Varian Cary Eclipse Fluorescence-Spectrophotometer (Agilent). The extinction coefficient ε_{max} was calculated according to Lambert-Beer for the absorbance maxima.

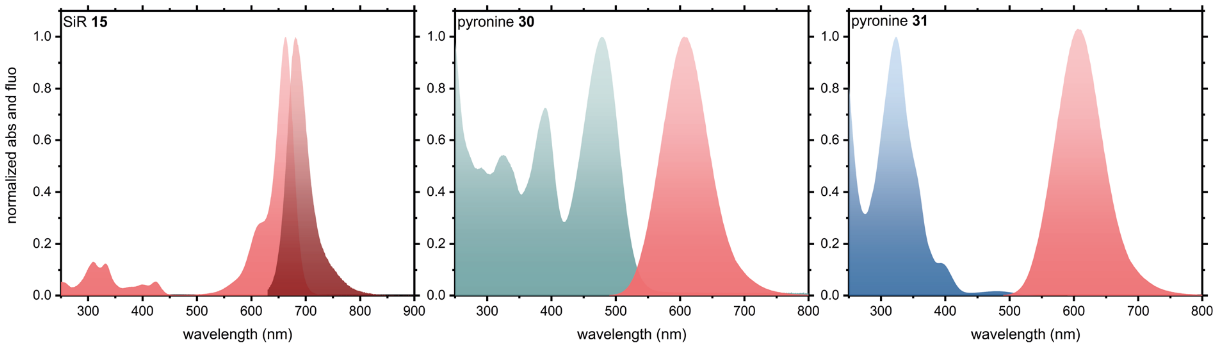

Figure S1: Normalized absorption (abs) and emission (fluo) spectra of SiR **15** (max. abs at 663 nm, max. fluo at 681 nm with excitation at 640 nm), pyronine **30** (abs at 323 nm/390 nm/478 nm, max. fluo at 607 nm with excitation at 480 nm) and pyronine **31** (abs at 323 nm/393 nm/480 nm, max. fluo at 599 nm with excitation at 480 nm) in MeCN.

4.2 Fluorescence quantum yield

The fluorescence quantum yields were determined either in absolute measurements using a Quantaurus-QY absolute PL quantum yield spectrometer (model C11347-12, Hamamatsu) according to the manufacturer's instructions or in relative measurements according to the literature procedure[3,4] using the reference standard dye Nile blue in 5% (v/v) 0.1 M HCl in EtOH (ϕ_{ref} = 0.27)[5]. For relative measurements, the dye stocks were diluted in MeCN such that the absorbance ranged between 0.01 and 0.1 at the excitation wavelength of 600 nm.

Absorbance of each dilution was recorded in the 200-800 nm interval. Afterwards, emission of the same sample was recorded with excitation at 600 nm in the 610-800 nm interval. All dilutions were measured using the same parameters. All measurements were performed in

[3]C. Wurth et al., *Nat. Protoc.*, **2013**, 8, 8, 1535–1550.
[4]A. T. R. Williams, S. A. Winfield, J. N. Miller, *Analyst* **1983**, *108,* 1290, 1067–1071.
[5]R. Sens, K. H. Drexhage, *Journal of Luminescence* **1981**,. *24-5,* 709–712.

air-saturated solvents at ambient temperature. Absorbance at 600 nm was plotted versus the integrated fluorescence intensity and fitted by linear regression. The fit values of the slopes were used to calculate the fluorescence quantum yield according to

$$\phi = \phi_{ref}\left(\frac{m}{m_{ref}}\right)\left(\frac{n}{n_{ref}}\right)^2$$

with ϕ (ϕ_{ref}) being the fluorescence quantum yield, m (m_{ref}) the slope of the linear fit and n (n_{ref}) the refractive index of the solvent of the sample (reference standard).

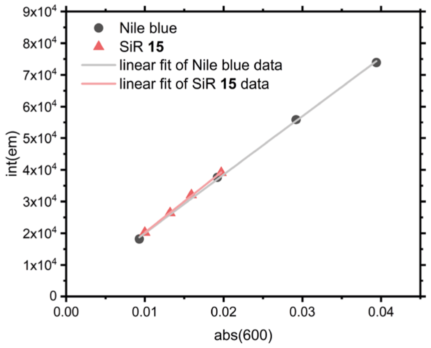

Figure S2: Relative measurement of the quantum yield of SiR **15** in MeCN ($\boldsymbol{n}$=1.34) using Nile blue as reference standard in 5% (v/v) 0.1 M HCl in EtOH ($\boldsymbol{n_{ref}}$=1.36).

Table S1: Fit Parameter for the relative measurement of the quantum yield of SiR **15** in MeCN using Nile blue as reference standard.

	SiR **15** in MeCN	Nile Blue
Equation	$y = a + mx$	
Weight	No Weighting	
Intercept a	716.70 ± 466.34	1517.19 ± 818.08
Slope m	1953490 ± 30831	1848437 ± 30594
Residual Sum of Squares	96537	941642
Pearson's r	0.99975	0.99973
R-Square (COD)	0.9995	0.99945
Adj. R-Square	0.99925	0.99918
